# Phytochemical Profiling of Processed Açaí Pulp (*Euterpe oleracea*) Through Mass Spectrometry and Its Protective Effects Against Oxidative Stress in Cardiomyocytes and Rats

**DOI:** 10.3390/antiox14060642

**Published:** 2025-05-27

**Authors:** Jefferson Romáryo Duarte da Luz, Eder Alves Barbosa, Rubiamara Mauricio de Sousa, Maria Lúcia de Azevedo Oliveira, Marcela Fabiani Silva Dias, Ingrid Reale Alves, Gisele Custódio de Souza, Elenilze Figueiredo Batista Ferreira, Carla Guzmán-Pincheira, Maria das Graças Almeida, Gabriel Araujo-Silva

**Affiliations:** 1 Organic Chemistry and Biochemistry Laboratory, Amapá State University (UEAP), Av. Presidente Vargas, s/n, –Centro, Macapá 68900-070, AP, Brazil; jefferson.luz@ueap.edu.br (J.R.D.d.L.); elenilze.batista@ueap.edu.br (E.F.B.F.); 2 Department of Biological and Health Sciences–DCBS, Federal University of Amapá (UNIFAP), Rodovia Rod. Josmar Chaves Pinto, Macapá 68903-419, AP, Brazil; marceladiazunifap@gmail.com (M.F.S.D.); ingridreale10@gmail.com (I.R.A.); gi.custodio.souza@gmail.com (G.C.d.S.); 3 Laboratory of Synthesis and Analysis of Biomolecules (LSAB), Institute of Chemistry, Darcy Ribeiro, University of Brasilia, Brasília 70910-900, DF, Brazil; bioederr@gmail.com; 4 Multidisciplinary Research Laboratory, DACT, Health Sciences Center, Federal University of Rio Grande do Norte, R. Gen. Gustavo Cordeiro de Farias, s/n–Petrópolis, Natal 59012-570, RN, Brazil; rubiamaramds1@gmail.com (R.M.d.S.); maluciaaazevedo@gmail.com (M.L.d.A.O.); mgalmeida84@gmail.com (M.d.G.A.); 5 Faculty of Health Care Sciences, San Sebastián University, Lientur 1457, 3° Piso, Edificio Los Alerces, Concepción 5110693, Chile; carla.guzman@uss.cl

**Keywords:** *Euterpe oleracea* Mart., açaí pulp, Amazon (Brazil), antioxidant properties, oxidative stress, cardiovascular diseases

## Abstract

The antioxidant capacity and modulation of oxidative stress by industrially processed açaí pulp extract from the Amazon (APEA) and its major anthocyanins, cyanidin 3-glucoside (C3G) and cyanidin-3-O-rutinoside (C3R), were evaluated as potential strategies for preventing cardiovascular diseases. The APEA was chemically characterized using ultrafast liquid chromatography-mass spectrometry (UFLC-MS), which revealed six main phenolic compounds. Notably, 9-(2,3-dihydroxypropoxy)-9-oxononanoic acid, acanthoside B, roseoside, cinchonine, and nonanedioate were identified for the first time in açaí extracts. In vitro antioxidant assays demonstrated that APEA exhibited strong DPPH- and ABTS-radical-scavenging activities (up to 80% inhibition and 65 mmol TE/100g DW, respectively) and showed ferrous- and copper-ion-chelating activities comparable to those of EDTA-Na_2_ at higher concentrations (up to 95% inhibition). Hydroxyl and superoxide radical scavenging activities reached 80% inhibition, similar to that of ascorbic acid. In H_2_O_2_-treated H9c2 cardiomyocytes, APEA significantly reduced the intracellular ROS levels by 46.9%, comparable to the effect of N-acetylcysteine. APEA also attenuated menadione-induced oxidative stress in H9c2 cells, as shown by a significant reduction in CellROX fluorescence (*p* < 0.05). In vivo, APEA (100 mg/kg) significantly reduced CCl-induced hepatic lipid peroxidation (MDA levels), restored glutathione (GSH), and increased the antioxidant enzymes CAT, GPx, and SOD, demonstrating superior effects to C3G and C3R, especially after 21 days of treatment (*p* < 0.001). These findings suggest that Amazonian açaí pulp (APEA) retains potent antioxidant activity after industrial processing, with protective effects against oxidative damage in cardiomyocytes and hepatic tissue, highlighting its potential as a functional food ingredient with cardioprotective and hepatoprotective properties.

## 1. Introduction

Scientific interest in polyphenols increased after the “French paradox” highlighted the cardioprotective effects of diets rich in phenolic compounds, such as the Mediterranean diet, which emphasizes fruits, vegetables, fish, and moderate red wine consumption. [1,2]. Among dietary polyphenols, anthocyanins are water-soluble pigments that contribute to the red, purple, and blue coloration of many fruits and vegetables [3]. A well-known example is the fruit of the açaí palm (*Euterpe oleracea* Mart.), which contains an average of 440 mg of anthocyanins per kilogram of fruit [4]. Native to the Amazon region of tropical Central and South America, including Brazil, Ecuador, and Venezuela, this species belongs to the Arecaceae (palm) family.

To meet the growing global demand, Brazil has become the leading producer and exporter of açaí [5]. Its pulp is widely consumed locally and exported globally, particularly in processed forms, such as frozen pulp, juice, or powder [5]. Several studies have described the antioxidant activity of fresh açaí extracts, showing that they can reduce lipid peroxidation, inhibit ROS production, and enhance the activity of antioxidant enzymes, such as superoxide dismutase (SOD), catalase (CAT), and glutathione peroxidase (GPx), in vitro and in vivo [5,6,7,8].

Moreover, chromatographic analyses of fresh açaí pulp have identified various phenolic constituents, including flavonoids, proanthocyanidins, and anthocyanins, particularly cyanidin derivatives such as cyanidin 3-glucoside (C3G) and cyanidin-3-O-rutinoside (C3R) [9,10,11,12,13]. However, few studies have investigated the chemical composition of açaí pulp after industrial processing, and even fewer have compared its biological efficacy with that of its isolated components. Industrial processing can alter the chemical structure of bioactive compounds, potentially affecting their stability and bioactivity, which is a critical consideration for exported or shelf-stable açaí products [5,14].

Açaí berries are rich in diverse bioactive compounds, particularly anthocyanins, anthocyanidins, and other polyphenols, known for their ability to neutralize reactive oxygen species (ROS) [15]. These free radicals can damage key biomolecules, such as DNA, proteins, and lipids, leading to oxidative stress, which is a major contributor to various physiological and pathological conditions [16,17,18,19,20].

Under pathological conditions, oxidative processes alter various subcellular structures, including the sarcolemma, mitochondria, sarcoplasmic reticulum, and nucleus. For instance, reactive oxygen species (ROS) may influence myocardial contractility by oxidizing elements such as sarco/endoplasmic reticulum Ca^2+^-ATPase and key contractile proteins, such as tropomyosin and actin, resulting in impaired contractile function [15]. Elevated ROS levels have been reported in multiple cardiac pathologies, including myocardial fibrosis [21], cardiac hypertrophy [22], heart failure [23,24], and myocardial infarction [25]. Cardiovascular disorders are among the primary causes of death worldwide. Despite the availability of effective pharmacological treatments, their adverse effects have driven a growing interest in natural compounds, especially phytochemicals, such as anthocyanins, polyphenols, and flavonoids, as complementary strategies for prevention and therapy [26,27,28].

Although açaí berries are rich in secondary metabolites, especially anthocyanins and other phenolic compounds with high antioxidant capacity, and the Brazilian Amazon region is the largest producer of these fruits, transporting the frozen pulp to more distant regions of the Amazon biome can alter its chemical composition, as well as its capacity to reduce oxidative stress and, consequently, its potential to prevent cardiovascular diseases [5,20]. Importantly, despite its recognized antioxidant potential, there are limited data on the effect of processed açaí pulp on oxidative stress in cardiovascular or hepatic models, particularly using standardized cell-based and in vivo systems. This limits our understanding of its potential as a functional food or nutraceutical for mitigating the oxidative damage associated with chronic diseases. In this study, we aimed to address this gap by chemically characterizing the phenolic profile of industrially processed açaí pulp extract (APEA) using ultra-fast liquid chromatography coupled with mass spectrometry (UFLC-MS), identifying previously unreported compounds in açaí, and comparing the antioxidant effects of APEA with its major anthocyanins (C3G and C3R) in vitro using H9c2 cardiomyocytes and in vivo in a model of carbon tetrachloride (CCl_4_)-induced oxidative liver injury in rats. This integrative approach allows us to evaluate both the chemical integrity and the biological relevance of processed açaí pulp, contributing new insights into its cardioprotective and hepatoprotective potential.

## 2. Materials and Methods

### 2.1. Obtaining Industrially Processed Açaí Pulp Consumed in the Amazon/Brazil and Exported Worldwide

The freeze-dried açaí pulp used in this research was obtained from the Cooperative of Agroextractive Producers of Bailique and Beira Amazonas (AMAZONBAI), located at Passarela Rio Marinheiro, 671, Vila Progresso, Arquipélago do Bailique 68913-000, Macapá–AP, with an agroindústria facility based in the Macapá industrial district–Macapá-Mazagão Road–Matapi River. This açaí pulp has triple certification: FSC^®^ and Rainforest Alliance^TM^, Vegan Product Seal, and Amapá Seal–Product from the Middle of the World, which promotes the appreciation of goods produced in the State of Amapá, especially those with incentives from the Green Free Zone, which confers to extreme quality açaí pulp, 100% from native açaí groves and managed through community-based practices. The freeze-dried açaí pulp was reconstituted and homogenized in water at a concentration of 2.5 mg/mL and then called Açaí Pulp Extract from the Amazon–APEA. In a subset of experiments, the effectiveness of the main bioactive components of fresh Açaí according to the literature, cyanidin 3-glucoside (C3G) (Sigma, cat# PHL89616, St. Louis, MO, USA) and cyandin-3-O-rutenoside (C3R) (Sigma, cat# PHL80577, St. Louis, MO, USA), were independently examined. A total of three independent batches of industrially processed açaí pulp were analyzed. These batches were obtained from different production lots to ensure consistency in the chemical profiles and biological activities of the APEA extracts.

### 2.2. Phytohemical Analysis via Ultrafast Liquid Chromatography Coupled to Mass Spectrometry (LC–MS/MS)

The dried samples were reconstituted and homogenized in water at a concentration of 2.5 mg/mL, subjected to an ultrasound bath for 30 min, and centrifuged at 13,000 rpm for 30 min. The supernatant was filtered through a 0.22 µm syringe filter and transferred to 1.8 mL vials for LC-MS analysis. Chromatographic analyses were carried out using a UPLC Acquity H-Class system (Waters, Milford, MA, USA) equipped with an ACQUITY UPLC HSS T3 column (1.8 µm, 2.1 × 100 mm). The system was coupled to a Maxis 4G mass spectrometer (Bruker Daltonics, Bremen, Germany) fitted with an electrospray ionization (ESI) source operated in both positive and negative ionization modes. Prior to initiating the analysis, the column was conditioned using a solution composed of 5% acetonitrile and 0.1% formic acid in water, with the column temperature stabilized at 40 °C. Subsequently, an automatic injection of 1 µL of the sample was carried out. The chromatographic separation employed a linear gradient ranging from 5% to 95% acetonitrile with 0.1% formic acid over a 10 min interval, at a constant flow rate of 0.4 mL/min, while maintaining the temperature at 40 °C. Spectra were acquired in positive and negative ionization modes, monitoring ions in the mass range of 60–1250 *m*/*z*. The acquisition was configured to fragment ions with an intensity greater than 500. In addition, data from the blank controls were acquired. Before analysis, the spectrometer was calibrated with 5 mM sodium formate (HCOONa). The same solution was injected before each acquisition to perform internal calibration.

The LC-MS/MS mass data were converted to mzXML format using MSConvert software (ProteoWizard Tools) (version 3.0.24310). Precursor *m*/*z* values were corrected using a free script available at https://github.com/elnurgar/mzxml-precursor-corrector (accessed on 8 February 2025). Modified mzXML files were submitted for analysis using the Molecular-Library Search-V2 tool (release_14 version), and the data were subsequently analyzed using the GNPS (Global Natural Product Social Molecular Networking) online platform (http://gnps.ucsd.edu) (accessed on 8 February 2025). To refine the dataset, peaks associated with ~17 Da were removed, as were the *m*/*z* values corresponding to molecules 2022, 27, and 1084, along with 13 of 17 precursor *m*/*z* values in the MS/MS spectra. Only the six most intense peaks within each 50 Da window of the spectrum were retained. Following this, the data were subjected to clustering using MS-Cluster, applying mass tolerances of 0.02 Da for precursor ions and 0.1 Da for MS/MS fragment ions to generate consensus spectra. Any consensus spectrum comprising fewer than two individual spectra was excluded from further analysis with the GNPS spectral libraries. Library spectra were filtered and aligned with the input data. During spectral matching, associations were retained when the library and network spectra achieved a similarity score above 0.85 and shared at least four matching fragment peaks. Compounds were considered putatively identified in the sample if their spectra exhibited four or more matching ions, with a cosine score of 1 indicating complete spectral identity and a score of 1.0 indicating no similarity.

### 2.3. DPPH-Free-Radical-Scavenging Activity

The DPPH-radical-scavenging activity was determined according to [13] with some modifications. For this assay, different concentrations (5, 10, 25, 50 and 100 µg/mL) of APEA, C3G, and C3R were added to a 96-well plate, followed by the addition of 0.03 mM DPPH ethanolic solution. The control consisted of only the ethanolic DPPH solution, and the blank consisted of the ethanolic solvent used to dissolve DPPH. The plates were then incubated for 30 min at room temperature in the dark. After incubation, the absorbance was measured using a microplate ELISA reader (Epoch-Biotek, Winooski, VT, USA) at a wavelength range of 517 nm. The percentage of radical scavenging activity (SR) was calculated using the following equation:

$$\%\mathrm{SR}=100-[\frac{\left(Abs\;control-Abs\;sample\right)}{\left(Abs\;control-Abs\;blank\right)}\times 100]$$

### 2.4. Determination of the ABTS-Free-Radical-Scavenging Ability

To generate the ABTS•+ radical, a 1:1 mixture of 7 mM ABTS (2,2′-azino-bis-(3-ethylbenzothiazolin-6-sulphonic acid)) and 2.45 mM potassium persulfate was prepared and left to incubate in the dark for 16 h. The resulting ABTS•+ radical solution was subsequently diluted at a ratio of approximately 1:10 using absolute ethanol or until it reached an absorbance of 0.7 at 754 nm. A stock solution of Trolox (2.5 nM) was used to prepare a calibration curve, with serial dilutions of 12.5%, 25%, 50%, and 75%. For sample analysis, 10 µL of APEA, C3G, or C3R was dispensed into a 96-well Tissue Culture Plate, followed by the addition of 200 µL of the ABTS•+ radical solution. The absorbance was measured at 270 nm using a microplate ELISA reader (Epoch-Biotek, Winooski, VT, USA). The antioxidant capacity determined via the ABTS•+ assay is reported in millimoles of Trolox equivalent per 100 g dry weight (mmol TE/100 g DW).

### 2.5. Ferrous-Ion-Chelating Assay

The iron ion (Fe^2+^)-chelating capacity of the samples was assessed using the ferrozine-based colorimetric assay. A series of six concentrations of ethylenediaminetetraacetic acid disodium salt (EDTA-Na_2_) ranging from 0 to 50 μg/mL, along with varying concentrations of APEA, C3G, and C3R, were distributed into the wells of a 96-well plate. This was followed by the addition of distilled water and a 0.3 mmol/L solution of ferrous sulfate (FeSO_4_). After a 5-min incubation period, ferrozine solution (0.8 mmol/L ferrozine solution was added, and the plate was incubated for an additional 10 min. A blank, consisting of either a sample or a standard combined with water, was used to correct the background coloration. The absorbance was recorded at 562 nm using a microplate ELISA reader (Epoch-Biotek, Winooski, VT, USA). The Fe^2+^ complex formation was subsequently calculated using the following formula:

$${\mathrm{Fe}}^{2+}\mathrm{chelating}\;\mathrm{ability}\;(\%)=[\frac{\left(Abs\;std\;0-Abs\;sample\right)}{\left(Abs\;std\;0\right)}\times 100]$$

### 2.6. Chelating Activity of Copper Ions

The copper ion (Cu^2+^)-chelating activity of the samples was evaluated using the pyrocatechol violet (PV) colorimetric assay. Six EDTA-Na_2_ standards and various concentrations of APEA, C3G, and C3R were added to 96-well plates. Subsequently, sodium acetate buffer (50 mmol/L, CH_3_COONa) at pH 6 and a 100 mg/L solution of copper(II) sulfate pentahydrate (CuSO_4_·5H_2_O) were added, followed by a 2 min incubation. Subsequently, a 2 mmol/L solution of pyrocatechol violet was introduced. To account for background coloration, blanks were prepared by mixing each sample or standard with sodium acetate buffer. The plate was then incubated for 20 min, and absorbance readings were taken at 632 nm using a microplate ELISA reader (Epoch-Biotek, Winooski, VT, USA). The percentage of Cu^2+^ complex formation was determined using the following equation:

$${\mathrm{Cu}}^{2+}\mathrm{chelating}\;\mathrm{ability}\;(\%)=[\frac{\left(Abs\;std\;0-Abs\;sample\right)}{\left(Abs\;std\;0\right)}\times 100]$$

### 2.7. Hydroxyl-Radical-Scavenging Activity

The hydroxyl-radical-scavenging capacity was assessed using the Fenton reaction [21]. APEA, C3G, and C3R were dissolved in phosphate-buffered saline and then combined with 10 mM FeSO_4_·7H_2_O-EDTA, 10 mM 2-deoxyribose, and 10 mM H_2_O_2_. The resulting mixture was incubated at 37 °C in the dark for 4 h. Following incubation, 1% thiobarbituric acid (TBA) and 2.8% trichloroacetic acid (TCA) solutions were added, and the samples were heated at 100 °C for 20 min. After cooling to room temperature, absorbance was measured at 490 nm using a microplate ELISA reader (Epoch-Biotek, Winooski, VT, USA). Ascorbic acid was used as the positive control. The hydroxyl-radical-scavenging activity was determined using the following formula:

$$\mathrm{Hydroxyl}\;\mathrm{radical}\;\mathrm{scavenging}\;\mathrm{activity}\;(\%)=[\frac{\left(Abs\;std\;0-Abs\;sample\right)}{\left(Abs\;std\;0\right)}\times 100]$$

### 2.8. Superoxide-Radical-Scavenging Assay

Superoxide-radical-scavenging activity was determined following the method described by Ewing et al. [22]. APEA, C3G, and C3R, previously diluted in water, were mixed with 0.1 M Tris-HCl buffer (pH 7.4), 100 μM phenazine methosulfate (PMS), 500 μM nitroblue tetrazolium (NBT), and 500 μM NADH. The mixtures were incubated at room temperature in the dark. After a 10 min incubation period, the absorbance was recorded at 560 nm using a microplate ELISA reader (Epoch-Biotek, Winooski, VT, USA). Ascorbic acid was used as a positive control. The superoxide-radical-scavenging capacity was calculated using the following formula:

$$\mathrm{Superoxide}\;\mathrm{radical}\;\mathrm{scavenging}\;\mathrm{activity}\;(\%)=[\frac{\left(Abs\;std\;0-Abs\;sample\right)}{\left(Abs\;std\;0\right)}\times 100]$$

### 2.9. Cell Culture and Animals

H9c2 cells (ATCC^®^ CRL-1446™) were sourced from the American Type Culture Collection (ATCC, Rockville, MD, USA). The cells were cultured in Dulbecco’s Modified Eagle’s medium (DMEM), supplemented with 10% fetal bovine serum and a combination of streptomycin (10,000 µg/mL) and penicillin (10,000 IU/mL). Cultures were maintained at 37 °C in a humidified incubator containing 5% CO_2_. For the in vivo assays, this project was submitted to the Animal Use Ethics Committee of the Federal University of Amapá, CEUA-UNIFAP-Brazil, and approved under protocol number 010/2021. In this study, male Wistar rats from the Health Sciences Center Vivarium of the Federal University of Rio Grande do Norte, CCS-UFRN-BRAZIL, were used at 60 days of age and weighing 160–210 g. Animals were kept in collective cages (n = 5) under controlled lighting conditions (12 h light/dark cycles) and temperatures (22 ± 2 °C) with food and water ad libitum.

### 2.10. Cell Viability Assays

Rat myocardial H9c2 cells (1 × 10^4^ cells per well) were seeded into 96-well microplates and incubated for 24 h at 37 °C in a humidified atmosphere containing 5% CO_2_ to allow cell adhesion. Following this period, the cells were exposed in triplicate to APEA, C3G, and C3R at concentrations of 25, 50, and 100 μg/mL and incubated for an additional 4 h at 37 °C. After this treatment, 10 μL of MTT solution (5 mg/mL) was added to each well, followed by incubation under the same conditions. Subsequently, the culture medium was removed, and 100 μL of DMSO was added to each well to solubilize the formazan crystals, and cell viability was assessed at 570 nm. The cytotoxic effects of APEA, C3G, and C3R were also analyzed in triplicate using the Alamar Blue^®^ assay, under the same culture and exposure conditions. After treatment, 10 μL of Alamar Blue^®^ (400 μg/L) diluted in PBS was added to each well, and plates were incubated for 24 h at 37 °C. The reduction of Alamar Blue was monitored by measuring the absorbance at 570 and 600 nm using a microplate ELISA reader (Epoch-Biotek, Winooski, VT, USA). Cells maintained in DMEM without treatment served as the negative control [29,30]. APEA, C3G, and C3R were dissolved in Dulbecco’s modified DMEM medium.

### 2.11. Evaluation of Indicators of Apoptosis Through Incubation with DAPI

Rat myocardial H9c2 cells (35.55 × 10^4^ cells per well) were cultured on 13 mm circular coverslips in a 24-well plate at 37 °C for 45 min. Subsequently, DMEM supplemented with 10% fetal bovine serum (FBS) was added to reach a final volume of 1 mL, and the system was incubated in a humidified atmosphere containing 5% CO_2_ at 37 °C for 24 h. After incubation, the medium was removed, and the cells were cultured in serum-free medium for an additional 24 h. Cells were then treated individually with medium containing APEA, C3G, and C3R at a concentration of 100 μg/mL. Following treatment, cells were washed with cold PBS, fixed in 4% paraformaldehyde for 20 min, and permeabilized with 0.1% Triton X-100 for approximately 20 min. After further washing with PBS, cells were incubated with 4′,6-diamidino-2-phenylindole (DAPI) at a concentration of 1 mg/mL for 30 min at room temperature in the dark. The cells were then examined via fluorescence microscopy using a 330–380 nm fluorescence filter (OLYMPUS BX41 fluorescence microscope) [31]. APEA, C3G, and C3R were dissolved in Dulbecco’s modified DMEM medium.

### 2.12. Intracellular ROS Assay

ROS generation was assessed using 2′,7′-dichlorofluorescein diacetate (DCFH-DA). Upon entry into the cells, DCFH-DA is hydrolyzed by intracellular esterases to form dichlorofluorescein (DCFH), which is trapped within the cell. This nonfluorescent molecule is then oxidized by cellular oxidants to produce the fluorescent compound dichlorofluorescein (DCF). Briefly, H9c2 cells (1 × 10^4^ cells per well) were seeded in 96-well plates and incubated for 24 h. Afterward, the cells were treated with APEA, C3G, and C3R, both alone and in combination with hydrogen peroxide (H_2_O_2_, 500 μM), for an additional 24 h. Following treatment, the cells were incubated with serum-free medium containing 10 μM DCFH-DA (Sigma) at 37 °C in the dark for 2 h. The fluorescence generated by the dye was measured using a multi detection reader (BioTek Instruments Inc., Winooski, VT, USA) at excitation and emission wavelengths of 485 and 530 nm, respectively. The treated cells were observed directly under a fluorescence microscope (Olympus, Tokyo, Japan) at 100× magnification [32]. APEA, C3G, and C3R were dissolved in Dulbecco’s modified (DMEM) medium.

### 2.13. Oxidative Stress Assay

To assess cellular oxidative stress, CellROX^TM^ Green Assay (Invitrogen, cat. #C10444, Waltham, MA, USA), according to the manufacturer’s protocol. Briefly, cells were plated in a 96-well plate and incubated with menadione (vitamin K_3_; Cayman Chemical, cat #15950) to induce oxidative stress in the presence or absence of APEA, C3G, and C3R at different concentrations as noted in the text. After the treatment period, CellROX™ dye was added to the cells and incubated for 30 min. After washing with PBS, the resulting fluorescence, which was excited at 485 nm, was measured at an emission wavelength of 520 nm using a microplate fluorescence reader. The canonical antioxidant N-acetylcysteine (1 mM) was used as a positive control in all experiments to verify the accuracy and reliability of the results.

### 2.14. Carbon-Tetrachloride-Induced Oxidative Damage (CCl_4_) Assay

The rats were randomly assigned to eight groups with four animals per group as outlined below. Carbon tetrachloride (CCl_4_) was administered intraperitoneally (i.p.) to animals in groups 2–8, in a single dose of 2.5 mL/kg, diluted in corn oil (50% *v*/*v*). Group 1: normal control rats, received 1.25 mL/kg i.p. of the vehicle i. p. Group 2: toxicant treatment with a single dose of CCl_4_ (2.5 mL/kg, i.p.). Group 3: CCl_4_ treatment followed by APEA for 7 days (100 mg/kg, p.o.). Group 4: CCl_4_ treatment followed by C3G for 7 days (100 mg/kg, p.o.). Group 5: CCl_4_ treatment followed by C3R for 7 days (100 mg/kg, p.o.). Group 6: CCl_4_ treatment followed by APEA for 21 days (100 mg/kg, p.o.). Group 7: CCl_4_ treatment followed by C3G for 21 days (100 mg/kg, p.o.). Group 8: CCl_4_ treatment followed by C3R for 21 days (100 mg/kg, p.o.). APEA, C3G, or C3R was administered daily via gavage for 7 or 21 days, starting 24 h after CCl_4_-induced liver injury.

#### 2.14.1. Sample Collection and Processing for Biological Tests

Twenty-four hours after the final treatment, the animals were anesthetized intraperitoneally with a xylazine–ketamine mixture (1:1) and euthanized. Laparotomy was performed for pooled blood collection via cardiac puncture, followed by evisceration. Serum samples from each experimental group were pooled for a subsequent biochemical analysis to quantify creatinine, direct bilirubin (DB), urea, aspartate aminotransferase (AST), alanine aminotransferase (ALT), gamma-glutamyl transferase (γ-GT), and alkaline phosphatase (ALP). These measurements were carried out using commercial Labtest kits (Labtest^®^, Labtest Diagnóstica S.A., Lagoa Santa, Brazil), following the manufacturer’s instructions on an automatic LabMax Plenno automated biochemical analyzer (Labtest^®^, Labtest Diagnóstica S.A.). The livers were washed with 0.9% NaCl for a further evaluation of oxidative stress parameters according to Batista et al. [25]. All measurements were performed in triplicate.

#### 2.14.2. Preparation of Liver Homogenate

Liver tissue (1 g) from each experimental group was homogenized in cold 20 mM potassium phosphate buffer (pH 7.4) using a Potter–Elvehjem homogenizer to obtain a 10% (*v*/*v*) homogenate, as outlined by Batista et al. [33], with modifications. The homogenate was then centrifuged at 4000 rpm for 4 min at 4 °C, and the supernatant was collected for the evaluation of glutathione (GSH), glutathione peroxidase (GPx), superoxide dismutase (SOD), catalase (CAT), and thiobarbituric acid-reactive substance (TBARS/MDA) levels.

#### 2.14.3. Oxidative Stress Analysis

Lipid peroxidation was assessed by measuring the thiobarbituric-acid-reactive substance (TBARS) concentration (μmol/L) at 553 nm, following the colorimetric method described by Yagi [34]. Glutathione (GSH) quantification (μmol/L) was performed at 420 nm, based on the method outlined by Beutler et al. [35]. Catalase (CAT) activity (IU/mg protein) was measured at 230 nm as described by Beutler et al. [36]. Superoxide dismutase (SOD) (IU/mg protein) and glutathione peroxidase (GPx) (IU/mg protein) activities were determined at 510 and 340 nm, respectively, using a commercial Randox kit (Randox Laboratories Ltd., Crumlin, UK).

### 2.15. Statistical Analysis

The data were initially assessed for normality using the Shapiro–Wilk test. The results from each experiment were compared with their respective control groups using a Student’s *t*-test, followed by ANOVA and the post hoc Tukey–Kramer test. The variables were expressed as the mean ± standard deviation, and a *p*-value of less than 0.05 (*p* < 0.05) was considered statistically significant. All statistical analyses were performed using GraphPad Prism software (version 5.0; GraphPad Software, San Diego, CA, USA).

## 3. Results

**Figure 1:**
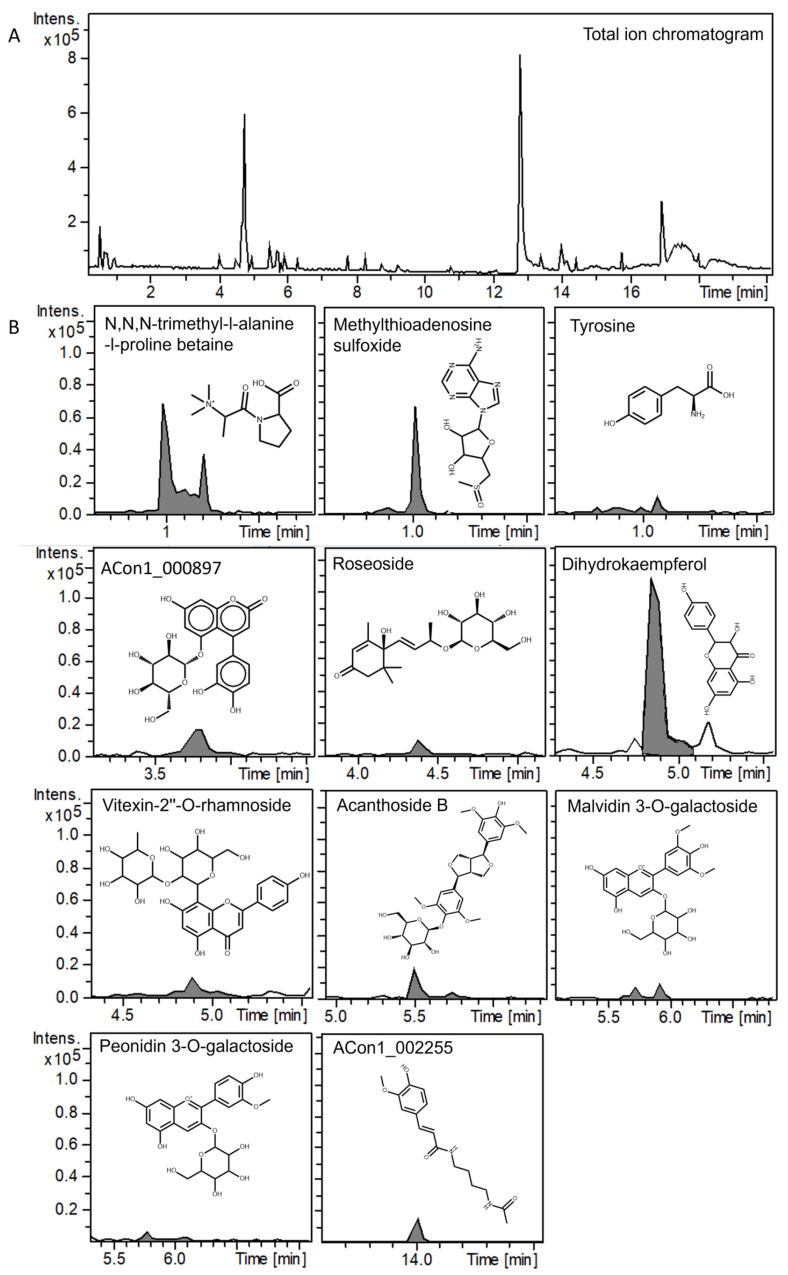
LC–MS/MS fingerprint of açaí pulp extract from the Amazon (APEA) in positive mode. (**A**) total ion chromatogram. (**B**) phytochemical profile of APEA. The comparison between library GNPS and query spectra of phytocomponents identified in Açai Pulp via LC-MS/MS analyses can be visualized in the Supplementary Materials.

**Figure 2:**
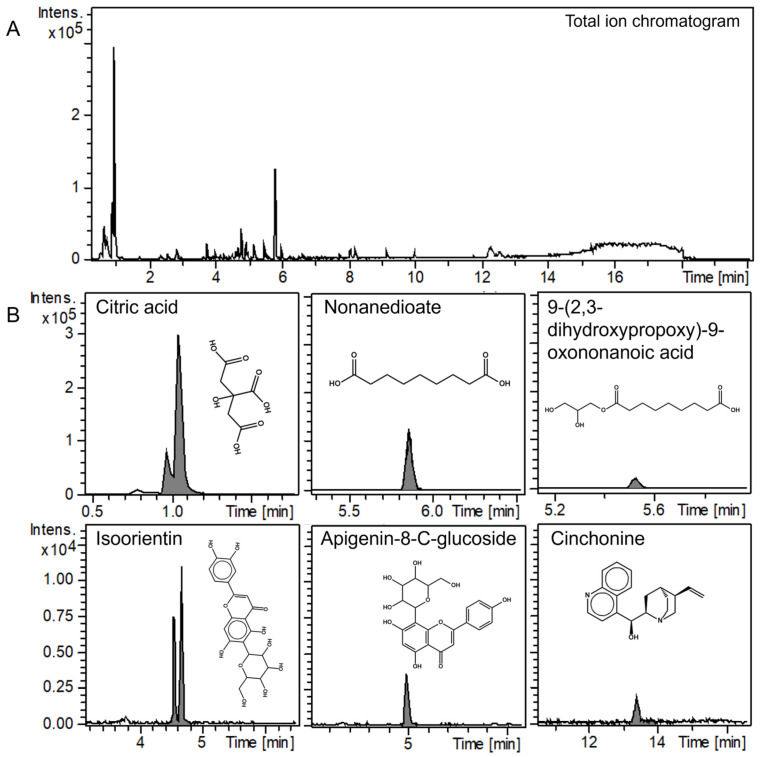
LC–MS/MS fingerprint of açaí pulp extract from the Amazon (APEA) in negative mode. (**A**) total ion chromatogram. (**B**) phytochemical profile of APEA. The comparison between library GNPS and query spectra of phytocomponents identified in açai pulp via LC-MS/MS analyses can be visualized in Supplementary Materials.

The LC-MS/MS analysis of industrially processed APEA was performed in both positive/negative ion modes to investigate its chemical composition, with a particular focus on polyphenols given their known presence in the extract. The MS/MS spectra of the identified compounds were putatively assigned using the GNPS database, considering only spectra with a cosine score ≥ 0.85 and a mass difference ≤ 0.005. Figure 1 presents the phytochemical profile of APEA in positive ion mode, revealing eleven chromatographic peaks that were identified and acquired. Figure 2 shows the phytochemical profile of APEA in negative ion mode, showing six identified and acquired chromatographic peaks. Both ion modes corresponded to potential structures recorded in the GNPS database and were identified based on the analysis of fragment peaks generated via mass spectrometry. Table 1 provides the details of the putatively identified phytocomponents, including their corresponding cosine scores, mass differences, and masses. The identification of compounds in *Euterpe oleracea* is novel, as a 9-(2,3-dihydroxypropoxy)-9-oxononanoic acid, acanthoside b, roseoside, cinchonine, and non-anedioate, beyond the compounds vitexin-2″-O-rhamnoside, isoorientin, malvidin 3-O-galactoside, peonidin 3-O-galactoside, alkaloids, coumarins, and some bulky amino acids (e.g., tyrosine) or their isoforms. 

**Figure 3:**
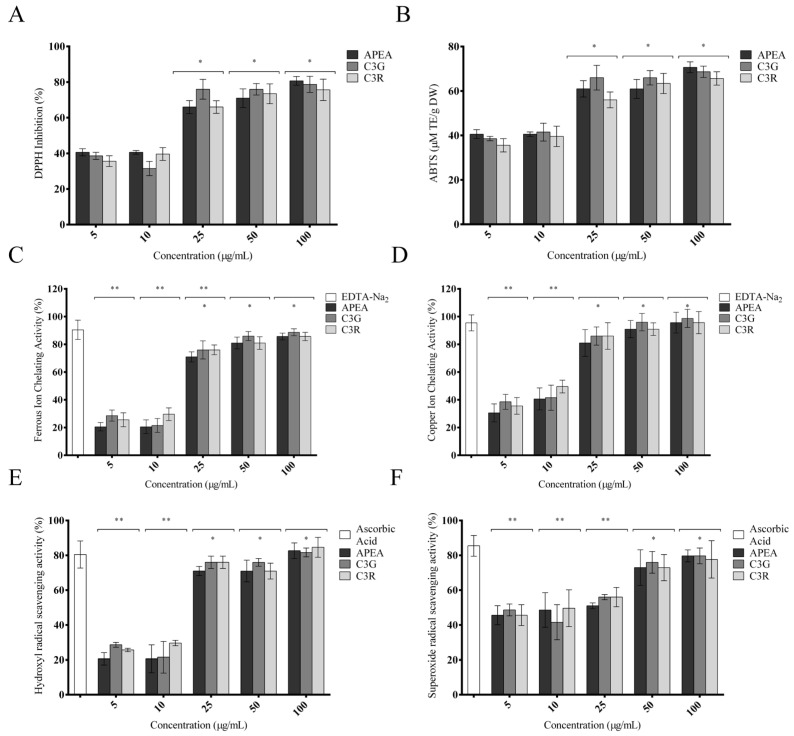
Antioxidant capacity of açaí pulp extract from the Amazon (APEA), cyanidin 3-glucoside (C3G), and cyandin-3-O-rutenoside (C3R). DPPH-free-radical scavenging activity (**A**); ABTS-scavenging activity (**B**); ferrous-ion-chelating activity (**C**); copper-ion-chelating activity (**D**); hydroxyl-radical-scavenging activity (**E**); and superoxide-radical-scavenging activity (**F**). Data represent the mean ± S.E.M. from three independent experiments. One-way ANOVA followed by the post hoc Tukey’s test. * *p* < 0.05 between the concentrations of samples [25, 50, and 100 μg/mL vs. 5 and 10 μg/mL]; ** *p* < 0.05 vs. the control group (EDTA-Na2 and ascorbic acid as an standards).

Figure 3 summarizes the antioxidant properties of açaí pulp extract from the Amazon (APEA) including a DPPH assay for anti-free radical activity, ABTS-radical-scavenging activity, ferrous- and copper-ion-chelating assay, and hydroxyl- and superoxide-radical-scavenging activity at various concentrations (5–100 µg/mL). All assay results are expressed based on a percentage of the inhibitory concentration of the APEA compared to the majority components of açaí (*Euterpe oleracea* Mart.) pulp according to the literature, cyanidin 3-glucoside (C3G) and cyandin-3-O-rutenoside (C3R), which was used as the standard antioxidant; only the ABTS assay was demonstrated using millimoles of Trolox equivalent per 100 g dry weight (mmol ET/100 g DW).

The three samples exerted an antioxidant effect against DPPH radicals (Figure 3A), with the greatest effect at the highest concentrations (25, 50 and 100 μg/mL), between 60 and 80% of inhibition (*p* < 0.0001). No significant differences were observed between the açaí pulp extract and its major components, indicating that active constituents in APAE could impart hydrogen to a free radical and terminate the potential damaging entity, suggesting a synergistic or comparable activity among components. The ABTS results showed high ABTS-radical-scavenging capability (Figure 3B) in all samples tested in the highest concentrations between 55 and 65 μM of trolox/g dry weight.

Regarding metal-chelating capacity, as shown in Figure 3C, the ferrous-ion-chelating capacity of APEA, C3G, and C3R was concentration-dependent and there was no significant difference between APEA and any of the major components tested (*p* > 0.05). However, there was no statistically significant difference between the samples and the EDTA-Na_2_ used as a standard, at the higher concentrations (25, 50 and 100 μg/mL), achieving an inhibition rate between 70 and 90% (*p* < 0.05). Figure 3D shows the copper-chelating power of APEA and its major constituent chemicals. The Cu^2+^-chelating activity was determined based on the formation of a blue Cu^2+^–PV complex. EDTA-Na_2_ exhibited a percentage of copper chelation of approximately 95% (*p* < 0.001). APEA achieved inhibition rates of 80, 90%, and 95% (*p* < 0.001) in a dose-dependent manner, and among the major compounds studied, C3G achieved the best results, being, respectively, 85, 95, and 98% (*p* < 0.05), but there was no significant difference between the samples.

The hydroxyl-radical-scavenging capability of APEA, C3G, and C3R compared to that of ascorbic acid was very satisfactory, indicating approximately 70–80% (*p* < 0.001) activity, showing no significant differences from the standard antioxidant (Figure 3E). The superoxide-scavenging capacity of the APEA, C3G, and C3R was similar to ascorbic acid, reaching its best result at around 80% (*p* < 0.005) only for the last two concentrations tested (50 and 100 μg/mL). Nevertheless, the tested samples efficiently scavenged superoxide anion radicals (Figure 3F).

**Figure 4:**
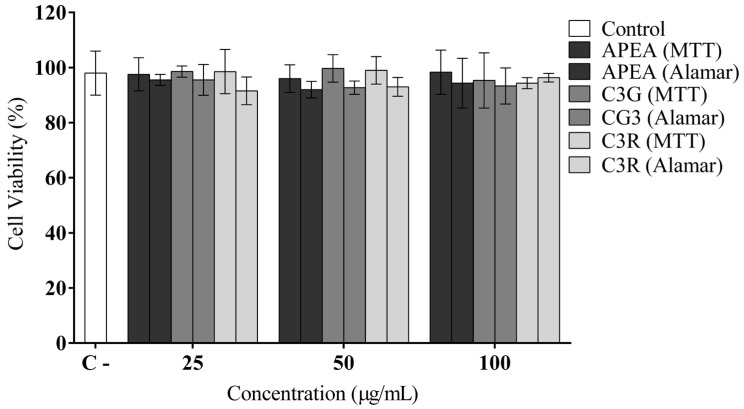
Cytotoxic effects of açaí pulp extract from the Amazon (APEA), cyanidin 3-glucoside (C3G), and cyandin-3-O-rutenoside (C3R) on the H9c2 cardiomyocyte cells line. Cell viability measured via MTT and Alamar Blue assays. DMEM culture medium was used as a negative control for cytotoxicity.

After the determination of the chemical composition and antioxidant capacity of açaí pulp extract from the Amazon (APEA), cyanidin 3-glucoside (C3G), and cyandin-3-O-rutenoside (C3R), their potential cytotoxic effects on the H9c2 cardiomyocytes cell line were evaluated (Figure 4). The results showed no cytotoxic effects evaluated, through cell viability assays using MTT and Alamar Blue^®^, after exposing cardiomyocyte cells to different concentrations of these substances. It is worth noting that concentrations of 25, 50, and 100 μg/mL were chosen from this point onwards as they presented greater antioxidant potential in previous experiments.

**Figure 5:**
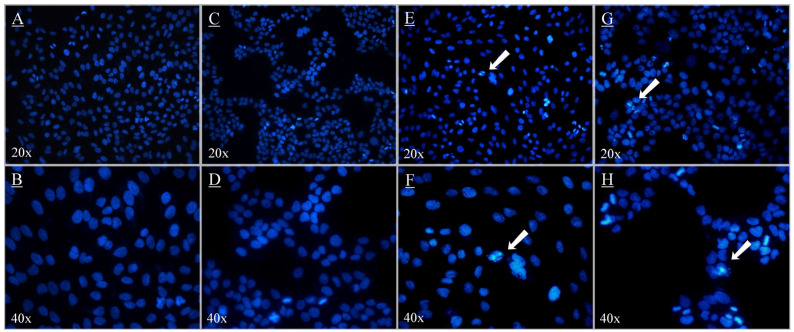
Micrograph of H9c2 cardiomyocyte cells treated with açaí pulp extract from the Amazon (APEA), cyanidin 3-glucoside (C3G), and cyandin-3-O-rutenoside (C3R). H9c2 cardiomyocyte cells were incubated with 100 µg/mL APEA, C3G, and C3R 24 h and labeled with DAPI to show the nuclear morphology. Control H9c2 cardiomyocyte cells without samples [negative control] (**A**,**B**); H9c2 cardiomyocyte cells showing no nuclear morphological changes, such as pyknosis and fragmentation, treated with APEA (**C**,**D**); H9c2 cardiomyocyte cells treated with C3G showing few signs of nuclear morphological changes (arrows) (**E**,**F**); H9c2 cardiomyocyte cells treated with C3R showing few signs of nuclear morphological changes (arrows) (**G**,**H**). (**A**,**C**,**E**,**G**) are micrographs seen with a 20× objective lens; (**B**,**D**,**F**,**H**) are micrographs seen with a 40× objective lens.

Nuclear morphological changes were assessed through DAPI staining. In the control group, the H9c2 cardiomyocytes appeared round and exhibited homogeneous staining (Figure 5). After 24 h of treatment, no blebbing nuclei, pyknotic bodies, morphological alterations, or granular apoptotic bodies were observed in either the control (Figure 5A,B) or APEA groups (Figure 5C,D). Minimal nuclear morphological changes were observed in the C3G and C3R groups (Figure 5E–H). The minimal morphological changes observed in H9c2 cardiomyocytes suggested that APEA, C3G, and C3R did not induce significant apoptosis, as there was little evidence of the blebbing of nuclei, pyknotic bodies, or other typical apoptotic features.

**Figure 6:**
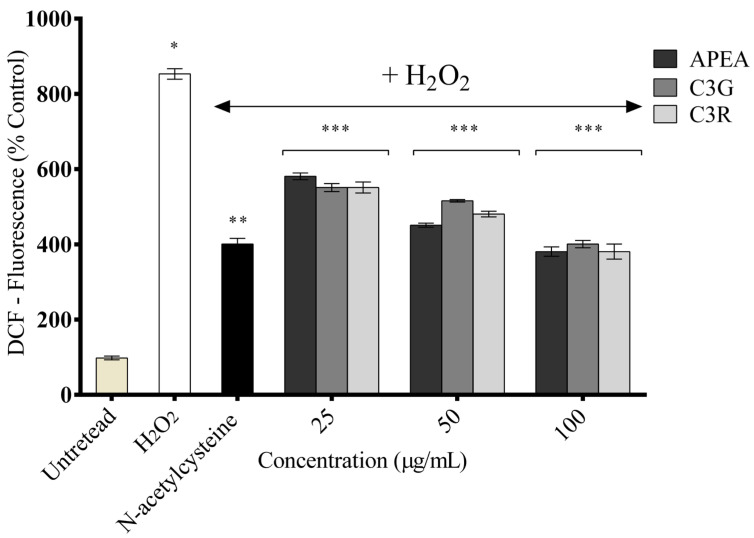
Intracellular reactive oxygen species (ROS) generation in the H_2_O_2_ and/or açaí pulp extract from the Amazon (APEA), cyanidin 3-glucoside (C3G), and cy-andin-3-O-rutenoside (C3R)-treated H9c2 cardiomyocyte cells. Data represent the mean ± S.E.M. from three independent experiments. One-way ANOVA followed by the post hoc Tukey’s test. * Difference between hydrogen peroxide and untreated group; ** difference between N-acetylcysteine and hydrogen peroxide; *** difference between samples and hydrogen peroxide.

A DCFH-DA assay was performed to evaluate the intracellular ROS-scavenging activities of APEA, C3G, and C3R in H_2_O_2_-treated H9c2 cardiomyocytes. After treatment with 500 μM H_2_O_2_ treatment, ROS generation increased by approximately 8.69 times in the H_2_O_2_-treated group compared with that in the untreated group (Figure 6). However, ROS generation in the treatment groups decreased in a dose-dependent manner at all concentrations tested (*p* < 0.001). In particular, the highest concentration of APEA, C3G, and C3R (100 μg/mL) showed the strongest ROS-scavenging activity, with reductions of 46.9%, 44.6%, and 46.2%, respectively, compared with that of the H_2_O_2_-treated cells (100%), with no statistical difference between the samples and the positive control group (N-acetylcysteine). These results indicate that APEA, C3G, and C3R treatment inhibited intracellular ROS generation in H_2_O_2_-treated H9c2 cardiomyocytes.

**Figure 7:**
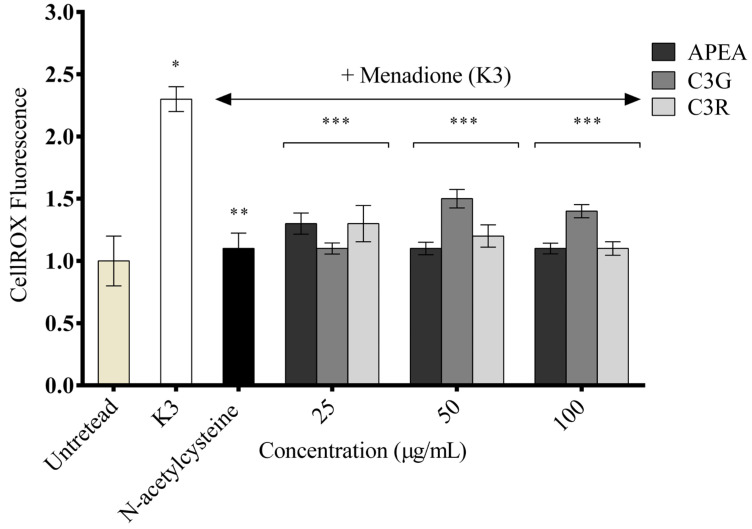
K3-induced oxidative stress in H9c2 cardiomyocyts cells with the CellROX^TM^ fluorescence assay. Açaí pulp extract from the Amazon (APEA), cyanidin 3-glucoside (C3G), and cyandin-3-O-rutenoside (C3R). Data represent the mean ± S.E.M. from three independent experiments. One-way ANOVA followed by the post hoc Tukey’s test. * Difference between K3 and untreated group, ** difference between N-acetylcysteine and K3, and *** difference between samples and K3.

To investigate the potential effects of APEA, C3G, and C3R on oxidative stress, H9c2 cardiomyocyte cells were treated for 1 h with APEA and its major components (25, 50, and 100 µg/mL) to determine if they had any effect on oxidative stress induced by menadione (also known as vitamin K_3_). The cells were also treated with 1 mmol/L N-acetylcysteine, a potent antioxidant. Oxidative stress was assessed using CellROX^TM^. As shown in Figure 7, the untreated group was not affected by CellROX^TM^ fluorescence in the absence of a stressor, whereas vitamin K_3_ induced a marked increase in CellROX^TM^ fluorescence. As expected, pretreatment with the potent antioxidant N-acetylcysteine significantly inhibited vitamin K_3_-induced oxidative stress (*p* < 0.001). All APEA, C3G, and C3R samples showed significantly reduced oxidative stress induced by K3 (*p* < 0.05), with no differences between the samples tested.

Since we demonstrated that APEA, C3G, and C3R display antioxidant activity in vitro and in cell systems, we treated rats with APEA, C3G, and C3R in an experimental model of oxidative stress to detect any changes in their biochemical and redox statuses. Oxidative stress was measured in the liver following CCl_4_-induced oxidative damage in rats. According to the results given in Table 2, serum levels of liver enzymes AST, ALT, γ-GT, and ALP showed that liver enzymes increased significantly in CCl_4_-damaged rats compared to the controls, as well as kidney markers (*p* < 0.05). The use of APEA, C3G, and C3R at a dose of 100 mg/kg prevented significant increases in AST, ALT, γ-GT, ALP, DB, urea, and creatinine (*p* < 0.05). Comparing all samples, APEA (21 days) showed a greater decrease in the biochemical parameters related to liver and kidney functions (*p* < 0.01).

The results for the hepatic antioxidant markers are summarized in Table 3. The data indicated that CCl_4_ significantly increased TBARS/MDA levels (a marker of lipid peroxidation in the liver) compared to the control group (*p* < 0.05). Treatment with 100 mg/kg APEA, C3G, and C3R significantly reduced lipid peroxidation (*p* < 0.05) compared to the CCl_4_ group. Notably, APEA demonstrated the greatest reduction in lipid peroxidation (*p* < 0.001) compared to C3G and C3R. Post-treatment with APEA, C3G, and C3R (100 mg/kg) for 7 and 21 days significantly improved the CCl4-induced depletion of GSH activity in the liver. Again, APEA showed superior activity (*p* < 0.001) compared to the other components tested as standards at both treatment periods (7 and 21 days).

An evaluation of the liver tissue enzymes CAT, GPx, and SOD showed that CCl_4_ significantly reduced the production of these enzymes compared to the controls, indicating damage to the liver (*p* < 0.05). The three samples tested in this study significantly prevented damage to the liver tissue and thus prevented a decrease in CAT in the groups that received APEA, C3G, and C3R compared to the control (*p* < 0.05). APEA presented higher activity than the majority of the compounds (*p* < 0.001) at both time points (7 and 21 days).

## 4. Discussion

Cardiovascular diseases are complex, multifactorial conditions and remain the foremost cause of mortality globally, as reported by the World Health Organization (WHO) [37]. Their pathophysiology includes a wide range of disorders, such as coronary artery disease, stroke, hypertension, heart failure, congenital heart defects, and various vascular conditions [15]. Oxidative stress is a key factor in the onset and progression of cardiovascular diseases, which occur when the balance between reactive oxygen species (ROS) and antioxidant defenses is disturbed. Elevated ROS levels can have various detrimental effects on the cardiovascular system, including endothelial dysfunction, which damages the lining of blood vessels and increases the risk of atherosclerosis and hypertension. In addition, ROS trigger chronic inflammation by activating proinflammatory pathways, further contributing to vascular damage [25,38].

Given the well-established role of oxidative stress in the development and progression of cardiovascular diseases, numerous studies have focused on evaluating the potential benefits of antioxidant-based therapies. Although a broad spectrum of therapeutic targets are being explored for the treatment of cardiovascular diseases, many medications continue to be the primary treatment options. These include statins, angiotensin-converting enzyme (ACE) inhibitors, angiotensin receptor blockers, calcium channel blockers, fibrates, alpha- and beta-blockers, diuretics, and mineralocorticoid receptor antagonists. It is essential to acknowledge that many of these drugs are associated with adverse effects in the human population [15,22,39,40,41,42].

Previous studies demonstrated that maintaining a balanced diet plays a crucial role in preventing cardiovascular disease [43]. Diets high in fruits and vegetables, such as the Mediterranean diet, are closely associated with increased life expectancy. Moreover, such diets help to reduce the occurrence of cardiovascular diseases [1,2], and epidemiological studies suggest that individuals who consume polyphenol-rich foods have a significantly lower risk of developing cardiovascular conditions [44,45,46,47]. This study is based on research indicating that açaí berry pulp is a rich source of polyphenols. It aims to present the chemical profile of industrially processed açaí pulp extract from the Amazon and explore its potential role in preventing cardiovascular disease, possibly through mechanisms that involve reducing oxidative stress.

In terms of the phytochemical composition, phenolic compounds stand out as the predominant class of secondary metabolites found in the Euterpe genus [48]. Based on this approach, industrially processed açaí (*Euterpe oleracea* Mart.) pulp analyses revealed the presence of six phenolic compounds: isoorientin, dihydrokaempferol, vitexin-2″-O-rhamnoside, apigenin-8-C-glucoside, malvidin 3-O-galactoside, and peonidin 3-O-galactoside. The last two compounds are anthocyanins found in açaí species. Açaí is widely recognized for its abundant bioactive compounds, with cyanidin 3-glucoside and cyanidin 3-rutinoside as its major anthocyanins. It also contains small amounts of other flavonoids, including apigenin, chrysin, epicatechin, luteolin, orientin, and vitexin [49]. However, to the best of our knowledge, some secondary metabolites found in this study are the first reported in the literature for *Euterpe oleracea*, such as 9-(2,3-dihydroxypropoxy)-9-oxononanoic acid, acanthoside B, roseoside, cinchonine, and non-anedioate, which exhibit antioxidant, immunomodulatory, and anti-inflammatory properties and can slow the progression of degenerative diseases [50,51,52,53,54].

The absence of the major anthocyanins cyanidin-3-arabinoside and cyanidin-3-arabinosyl-arabinoside in the freeze-dried açaí sample may be attributed to selective degradation during processing. The presence of galactosylated anthocyanins (e.g., malvidin 3-O-galactoside) and non-anthocyanin flavonoids (e.g., isoorientin) suggests potential structural rearrangements or the analytical prioritization of more stable flavonoid subclasses under the LC-MS/MS conditions employed [55,56,57].

The antioxidant properties observed in treatments using açaí pulp extract and its main fresh components are primarily associated with the presence of phenolic compounds. This suggests that these compounds play key roles in the inhibition of the synthesis and activity of various antioxidant mediators [16]. Therefore, the presence of these phytocomponents in APEA may be linked to the antioxidant effects observed in this study. APEA and its major fresh compounds, used as standard antioxidants, exhibited this in vitro effect, as evaluated by the inhibition of DPPH- for anti-free-radical activity, ABTS-radical-scavenging activity, ferrous- and copper-ion-chelating activity, and hydroxyl- and superoxide-radical-scavenging activity.

Numerous studies have demonstrated that phenolic compounds derived from plant materials have antiradical activity, reducing power, and protective effects against DNA damage caused by reactive oxygen species (ROS) [3,4,5,23,58]. Regarding the DPPH, ABTS, metal ion chelating, hydroxyl and superoxide radical scavenging, the results of this work demonstrated that açaí consumed in the Amazon presented an 80% inhibition of DPPH, from 55 to 65 μM of trolox/g DW in an ABTS assay, values between 70 and 95% of inhibition of ferrous and copper chelating, and approximately 80% of hydroxyl- and superoxide-radical-scavenging activity inhibition, which indicates a considerable antioxidant capacity. These values are in accordance with previous studies that suggest that açaí has a high antioxidant potential due to its rich composition of polyphenols, flavonoids, and anthocyanins [14,59].

Furthermore, polyphenols are known for their low cytotoxicity [4,59], which was corroborated by the results obtained in this study, particularly in relation to the viability of H9c2 cardiomyocytes. None of the analyzed samples caused significant toxicity in the two experimental models evaluated in this study.

In terms of APEA’s ability to reduce intracellular ROS production in H9c2 cardiomyocytes, the açaí extract demonstrated 45% inhibition compared to the H_2_O_2_ treatment used as the control. The excessive production of intracellular ROS can cause both direct and indirect damage to nucleic acids and alter the structure and function of cellular lipids and proteins, ultimately resulting in apoptosis and necrosis [60]. Oxidative stress, which arises from an imbalance between ROS accumulation and antioxidant defense, plays a crucial role in cellular damage [3,5,12,14]. Many studies have investigated the role of oxidative stress in the onset of various cardiovascular diseases [3,5,12,14]. As noted previously, the results of this study showed that H_2_O_2_ treatment significantly elevated intracellular ROS levels, while all concentrations of APEA and its main components effectively reduced ROS in H9c2 cardiomyocyte cells in a dose-dependent manner. These findings suggest that treatment with APEA, C3G, and C3R can effectively regulate ROS production, likely owing to their potent radical-scavenging abilities.

To assess whether APEA, C3G, and C3R can modulate oxidative stress, the H9c2 cardiomyocytes were analyzed via a CellROX assay using menadione/K3 to induce oxidative stress. CellROX Green is a fluorogenic probe that measures the oxidative stress in living cells. It is a cell-permeable dye that exhibits a weak fluorescence upon reduction. Upon oxidation by reactive oxygen species (ROS), it displays a vibrant, photostable green fluorescence [61]. APEA, C3G, and C3R significantly reduced K3-induced oxidative stress in H9c2 cells. Based on these experiments, it can be concluded that the decrease in the oxidative stress levels in the tested cells mediated by the APEA is one of the mechanisms that is responsible for modulating the oxidative stress of berries like açaí. These modulatory effects may be associated with metabolites tentatively identified in the extract, as polyphenols such as those found in berries could potentially benefit human health through their reported properties of inhibiting oxidative stress and regulating cardioprotective mechanisms [62]. Given its wide variety of cardioprotective bioactive compounds, açaí has been investigated as a potential therapeutic agent for cardiovascular diseases. Two studies explored the effects of malvidin, an anthocyanin found in red berries, and cyanidin-3-O-glucoside (C3G) in isolated rat hearts subjected to ischemia/reperfusion (I/R) injury. Pretreatment with malvidin (10^−10^ and 10^−6^ mol/L) or C3G (20 μM) led to an increase in left ventricular (LV) pressure and a significant reduction in both cell apoptosis and necrosis [63]. Louis et al. [64] investigated the in vitro effects of blueberry phenolic fractions (BF) in protecting adult rat cardiomyocytes from norepinephrine (NE)-induced damage. NE induced cell hypertrophy and death via calpain activation, but pre-treatment with BF (6.55 µg/mL) reduced calpain activation, apoptosis, and boosted the activity of antioxidant enzymes.

Recently, the effects of apigenin and vitexin, two compounds found in açaí species, on I/R injury were investigated in H9c2 and neonatal rat cardiomyocytes. Both apigenin (40 µM) and vitexin (10, 30, and 100 µM) significantly boosted cell viability, minimized ROS production, and reduced apoptosis and necrosis [65]. In addition, vitexin exhibited substantial protective effects against myocardial I/R injury in isolated rat hearts. When hearts were treated with vitexin (50, 100, 200 µM) for 20 min prior to ischemia, the treatment effectively prevented the I/R-induced decrease in coronary flow, improved histopathological conditions in the myocardium, lowered inflammatory cytokine levels, and inhibited apoptosis [65].

In a study conducted by Figueiredo et al. [8], male *Wistar* rats were administered açaí pulp to assess its impact on cardiac remodeling in animal models. The results showed that supplementation with açaí pulp after myocardial infarction improved energy metabolism, reduced oxidative stress (lower malondialdehyde levels, *p* = 0.023), and significantly attenuated cardiac remodeling post-infarction in a dose-dependent manner. Studies conducted by Monteiro et al. [66] and Vilhena et al. [67] reported similar findings, showing substantially reduced cardiovascular remodeling and recovered endothelial dysfunction in human umbilical vein endothelial cells (HUVECs) and rats, probably through its antihypertensive and antioxidant actions, which may also be associated with the phenolic groups detected in the chemical composition of the extract. Studies suggest that a combination of polyphenols exhibits greater biological activity than individual compounds [68].

In an in vivo model of oxidative stress measured in the liver (hepatotoxicity) from CCl_4_-induced oxidative damage, APEA, C3G, and C3R prevented an increase in biochemical parameters related to liver and kidney functions and modulated the liver tissue enzymes GSH, CAT, GPx, and SOD and lipid peroxidation induced by malondialdehyde in rats. After being stored in the liver, CCl_4_ undergoes biotransformation in microsomes via CYP2E1 enzymes, leading to the formation of the reactive radical CCl_3_•. This trichloromethyl radical interacts with oxygen to produce the even more reactive CCl_3_O_2_• [69]. Liver damage caused by CCl_4_ is mainly attributed to the induction of oxidative stress and lipid peroxidation [70,71].

During oxidative stress, toxic CCl_4_ metabolites deplete and impair antioxidant defenses (such as CAT, SOD, GPx, and GSH), while concurrently elevating pro-oxidant markers (including xanthine oxidase, NADPH oxidase, GSSG, and H_2_O_2_), thereby intensifying oxidative stress and contributing to liver cell injury. [72,73]. In the lipid peroxidation phase, unneutralized CCl_4_ radicals form covalent bonds with proteins and lipids in hepatocytes, mitochondria, and endoplasmic reticulum membranes. The reactive CCl_3_O_2_• radical extracts a hydrogen atom from unsaturated fatty acids in these membranes, generating a lipid radical that triggers the lipid peroxidation process, causing both morphological and functional damage to hepatocytes [72].

According to Popovic et al. [73], pre-treatment with bilberry fruit extract anthocyanins significantly reduced the serum activities of specific damage biomarkers (such as glutamate dehydrogenase and sorbitol dehydrogenase), thereby protecting hepatocyte membranes and organelles from the toxic effects of CCl_4_. Notably, there was a significant decrease in malate dehydrogenase activity, which, combined with histopathological findings, indicated a well-preserved periportal space and only mild damage to the centrilobular space, suggesting the hepatoprotective effects of the bilberry fruit extract. Machado et al. [74] showed that açaí frozen pulp when administrated to male rats daily for 14 days was able to prevent the increase of ALT and AST caused by CCl_4_ in the serum, as well as decrease TNF-α, IL-1b, and IL-18 levels (pro-inflammatory cytokines closely associated with oxidative stress). Another work of the same research group using the same CCl_4_ experimental model demonstrated that açaí frozen pulp prevents enhanced TBARS, carbonyl, and sulfhydryl levels, when non-enzymatic antioxidant defenses were analyzed, and prevented CAT and SOD alterations [75].

Overall, these findings reinforce the notion that açaí pulp extract from the Amazon and its main components possess a strong antioxidant effect, which is linked to the prevention of cardiovascular diseases. However, additional studies, particularly clinical trials, are needed to determine whether industrially processed açaí pulp extract from the Amazon can help prevent such diseases by modulating antioxidant markers.

This study has some limitations that should be acknowledged. The chemical identification of certain compounds in the açaí pulp extract was performed at a putative level based on accurate mass and fragmentation patterns, without confirmation through authentic analytical standards or NMR. The in vivo experiments were conducted with a relatively small sample size, although sufficient to achieve statistical significance, which may limit the generalizability of the findings. While the in vitro models provide valuable insights into cellular antioxidant responses, they cannot fully replicate the complexity of oxidative stress mechanisms in human physiology. Future studies involving targeted metabolomics, pharmacokinetics, and clinical validation are warranted to confirm and expand upon these results.

## 5. Conclusions

The present study underscores the promising potential of industrially processed açaí pulp as a functional food with antioxidant, hepatoprotective, and cardioprotective properties. The evidence supports the idea that, even after processing, açaí pulp retains bioactive compounds capable of modulating oxidative stress, an underlying factor in the development and progression of several chronic diseases, including cardiovascular and liver-related disorders. This finding is particularly relevant in the context of global food distribution, where fresh fruits are often unavailable and industrially processed alternatives are more accessible.

Moreover, the identification of a broad range of polyphenols and other phytochemicals not traditionally associated with fresh açaí highlights the complexity and diversity of its chemical composition. This also suggests that processing may lead not only to preservation but also to the formation or concentration of new bioactive metabolites. These insights open new avenues for research focused on optimizing industrial techniques to maintain or enhance the nutraceutical potential of native fruits.

In this context, the inclusion of açaí pulp in the regular human diet or in the development of nutraceutical supplements may represent a practical and culturally relevant approach to oxidative stress prevention and health promotion, particularly in populations at risk for non-communicable diseases. The findings of this study contribute to the growing body of evidence supporting the medicinal and nutritional value of Amazonian biodiversity.

## Figures and Tables

**Table 1:** Phytocomponents identified in açaí pulp extract from the Amazon (APEA) via LC-MS/MS analyses.

Phytocomponents Matched with GNPS Data Base	Cosine	MassDiff	RT (min)	Molecular Formula	SpecMz	Adduct	Classification
N,N,N-trimethyl-L-alanine-L-proline-betaine	0.92	0.0000	1.0	C_11_H_20_N_2_O_3_	229.1550	M + H	Amino acid derivative
Methylthioadenosine sulfoxide	0.87	0.0010	1.0	C_11_H_15_N_5_O_4_S	314.0910	M + H	Nucleotide derivative
Tyrosine	0.90	0.0000	1.0	C_9_H_11_NO_3_	182.0810	M + H	Amino acid
Citric Acid	0.84	0.0000	0.9	C_6_H_8_O_7_	191.0186	M − H	Food preservative
4-(3,4-Dihydroxyphenyl)-5-(beta-D-glucopyranosyloxy)-7-hydroxy-2H-1-benzopyran-2-one	0.82	0.0010	3.6	C_21_H_20_O_11_	449.1090	[M + H]+	Coumarins
Roseoside	0.84	0.0020	4.4	C_19_H_30_O_8_	387.2010	M + H	Apocarotenoids
Isoorientin	0.91	0.0070	4.5	C_21_H_20_O_11_	447.0930	M − H	Flavonoids
Dihydrokaempferol	0.93	0.0000	4.9	C_15_H_12_O_6_	289.0710	M + H	Flavonoids
Vitexin-2″-O-rhamnoside	0.84	0.0050	5.0	C_27_H_30_O_14_	579.1720	M + H	Flavonoids
Apigenin-8-C-glucoside	0.86	0.0000	5.0	C_21_H_20_O_10_	431.0980	M − H	Flavonoids
9-(2,3-dihydroxypropoxy)-9-oxononanoic acid	0.92	0.0000	5.5	C_12_H_22_O_6_	261.1340	[M − H]−	Fatty acids and conjugates
Acanthoside B	0.82	0.0010	5.6	C_28_H_36_O_13_	598.2510	M + NH4	Lignans
Malvidin 3-O-galactoside	0.96	0.0010	5.8	C_23_H_25_O_12_	493.1350	Cat	Flavonoids
Peonidin 3-O-galactoside	0.96	0.0010	5.8	C_22_H_23_O_11_	463.1250	Cat	Flavonoids
Nonanedioate	0.97	0.0000	5.8	C_9_H_14_O_4-2_	187.0980	[M − H]−	Fatty acids and conjugates
Cinchonine	0.81	0.0110	13.5	C_19_H_22_N_2_O	293.1770	[M − H]−	Tryptophan alkaloids
(E)-N-(4-acetamidobutyl)-3-(4-hydroxy-3-methoxyphenyl)prop-2-enamide	0.93	0.0553	14.0	C_16_H_22_N_2_O_4_	635.3600	[2M + Na]+	Ornithine alkaloids

RT = retention time.

**Table 2:** Biochemical parameters of açaí pulp extract from the Amazon (APEA), cyanidin 3-glucoside (C3G), and cyandin-3-O-rutenoside (C3R) in a carbon tetrachloride-induced oxidative damage (CCl_4_) assay.

Biochemical Parameters	Control (Corn Oil)	CCl_4_ (in Corn Oil)	CCl_4_ + APEA (7 Days)	CCl_4_ + C3G (7 Days)	CCl_4_ + C3R (7 Days)	CCl_4_ + APEA (21 Days)	CCl_4_ + C3G (21 Days)	CCl_4_ + C3R (21 Days)
ALT (U/L)	158.0 ± 2.1	621.5 ± 32.7 *	186.9 ± 3.33 *	185.7 ± 7.08 *	195.2 ± 9.54 *	196.6 ± 8.01 *^(^**^)^	295.8 ± 6.98 *	293 ± 6.01 *
AST (U/L)	215.0 ± 2.3	764.3 ± 31.7 *	210.2 ± 9.39 *	210.7 ± 8.98 *	200.9 ± 13.3 *	200.3 ± 5.35 *^(^**^)^	302.9 ± 8.54 *	300 ± 8.79 *
γ-GT (U/L)	0.20 ± 0.12	14.9 ± 1.05 *	0.10 ± 0.01 *	0.27 ± 1.01 *	0.29 ± 0.32 *	0.13 ± 0.01 *^(^**^)^	0.82 ± 0.06 *	0.72 ± 0.12 *
ALP (U/L)	110.2 ± 1.2	299.5 ± 2.6 *	100.4 ± 1.25 *	100.5 ± 1.24 *	115 ± 1.15 *	109 ± 0.01 *^(^**^)^	165 ± 1.02 *	161 ± 1.43 *
DB (mg/dL)	0.30 ± 0.01	1.9 ± 0.2 *	0.38 ± 0.01 *	0.30 ± 0.05 *	0.43 ± 0.05 *	0.44 ± 0.01 *	0.40 ± 0.05 *	0.38 ± 0.12 *
Urea (mg/dL)	46.2 ± 1.71	92.9 ± 1.54 *	45.7 ± 2.81 *	40.5 ± 4.02 *	45.2 ± 5.21 *	44.8 ± 5.41 *^(^**^)^	64.7 ± 3.81 *	60.2 ± 1.62 *
Creatinine (mg/dL)	3.70 ± 0.64	8.2 ± 0.70 *	3.2 ± 0.13 *	3.1 ± 0.01 *	3.1 ± 0.41 *	3.1 ± 0.01 *^(^**^)^	5.1 ± 0.05 *	5.1 ± 0.05 *

Results are expressed as the mean ± SD. Control group, treated with vehicle (corn oil). Comparisons between groups were performed using an analysis of variance and Tukey’s post hoc test. (n = 4); alanine aminotransferase enzymes (ALT), aspartate aminotransferase (AST), gamma-glutamyl transferase (γ-GT), alkaline phosphatase (ALP), direct bilirubin (DB). * *p* < 0.05 values are significant compared to the control and sample groups, compared to the CCl_4_ group. ** *p* < 0.01 values are significant when comparing among açaí pulp extract from the Amazon (APEA), cyanidin 3-glucoside (C3G), and cyandin-3-O-rutenoside (C3R).

**Table 3:** Oxidative stress parameters of açaí pulp extract from the Amazon (APEA), cyanidin 3-glucoside (C3G), and cyandin-3-O-rutenoside (C3R) in a carbon tetrachloride-induced oxidative damage (CCl_4_) assay.

Oxidative Stress Parameters	TBARS/MDA (μmol/L)	GSH (μmol/L)	CAT (IU/mg Protein)	GPx (IU/mg Protein)	SOD (IU/mg Protein)
Control (corn oil)	30.4 ± 3.1 *	7.5 ± 0.27 *	104.6 ± 1.8 *	18.8 ± 0.5 *	84.3 ± 3.7 *
CCl_4_ (in corn oil)	87.3 ± 1.3	3.4 ± 0.07 *	73.2 ± 1.3 *	4.1 ± 0.3 *	11.3 ± 0.5 *
CCl_4_ + APEA (7 days)	40.4 ± 0.12 *^(^**^)^	6.2 ± 0.05 *(**)	99.1 ± 0.01 *(**)	38.2 ± 2.51 *	88.19 ± 1.32 *
CCl_4_ + C3G (7 days)	57.2 ± 1.2 *	4.65 ± 0.6 *	79.4 ± 1.25 *	37.5 ± 1.64*	81.5 ± 1.15 *
CCl_4_ + C3R (7 days)	58.3 ± 0.01 *	4.97 ± 0.2 *	80.3 ± 0.01 *	38.3 ± 0.05*	84.43 ± 0.05 *
CCl_4_ + APEA (21 days)	36.2 ± 1.35 *(**)	7.9 ± 0.04 *(**)	105.7 ± 1.21 *(**)	35.5 ± 0.02*	145.2 ± 3.11 *(**)
CCl_4_ + C3G (21 days)	50.8 ± 2.64 *	4.9 ± 0.2 *	77.2 ± 0.14 *	38.1 ± 0.01*	84.1 ± 1.41 *
CCl_4_ + C3R (21 days)	53.7 ± 1.64 *	5.3 ± 0.7 *	78.2 ± 0.33 *	39.1 ± 0.01*	88.1 ± 0.91 *

Values are presented as the mean ± SD. (n = 4). Comparisons between groups were performed using analysis of an ANOVA and Tukey’s post hoc-test. Thiobarbituric-acid-reactive substances/malondialdehyde (MDA), reduced glutathione (GSH), catalase (CAT), glutathione peroxidase (GPX), and superoxide dismutase (SOD). * *p* < 0.05 values are significant compared to the control and sample groups, compared to the CCl_4_ group. ** *p* < 0.01 values are significant when comparing among açaí pulp extract from the Amazon (APEA), cyanidin 3-glucoside (C3G), and cyandin-3-O-rutenoside (C3R).

## Data Availability

The original contributions presented in this study are included in the article. Additional questions can be directed to the author Jefferson Romáryo Duarte da Luz (jefferson.luz@ueap.edu.br).

## Supplementary Materials

The following supporting information can be downloaded at: https://www.mdpi.com/article/10.3390/antiox14060642/s1, Comparison of library GNPS and query spectra of phytocomponents identified in *P. parvifolium* hydroalcoholic bark extract using LC-MS/MS analysis.
