# Supplementary material for: Phytochemical Profiling of Processed Açaí Pulp (Euterpe oleracea) Through Mass Spectrometry and Its Protective Effects Against Oxidative Stress in Cardiomyocytes and Rats
Source: Antioxidants (Basel). 2025 May 27;14(6):642. doi: 10.3390/antiox14060642 (PMC12189401; doi:10.3390/antiox14060642)

Supplementary material

Comparison between library GNPS and query spectra of phytocomponents identified in Açai Pulp by LC-MS/MS analyses

Comparison between library GNPS (bottom) and query spectra phytocomponents identified in **Açai Pulp** (top). The structure of the phytocomponent identified is represented.

N,N,N-trimethyl-l-alanine-l-proline betaine

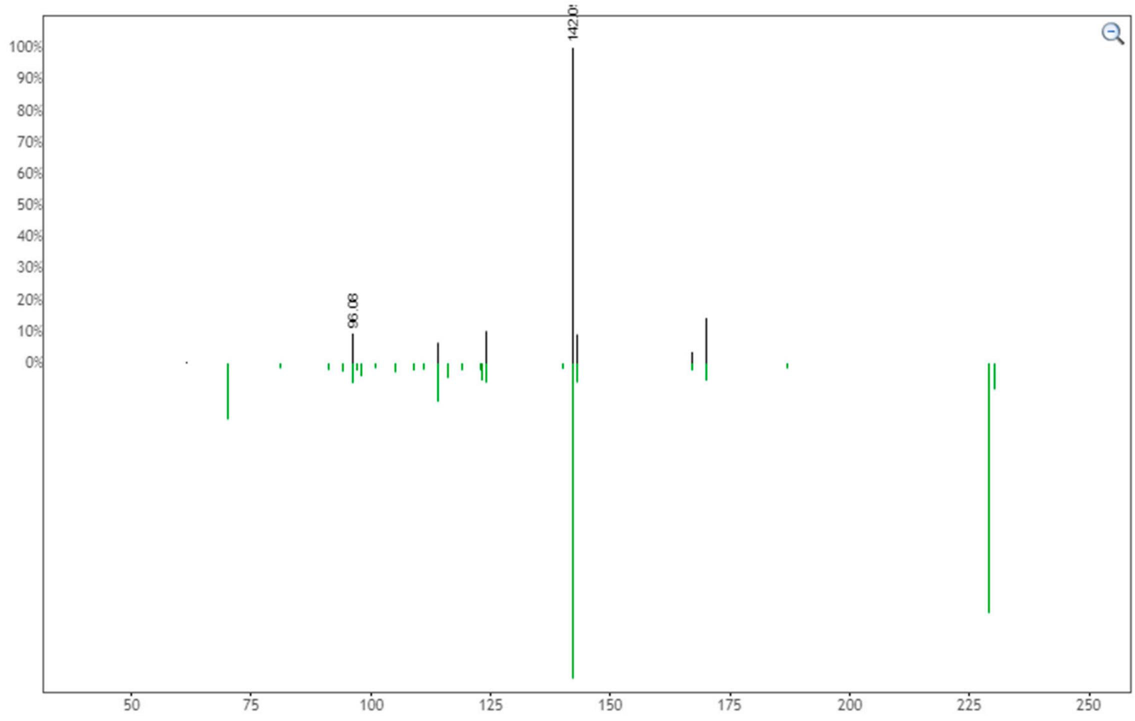

Methylthioadenosine sulfoxide

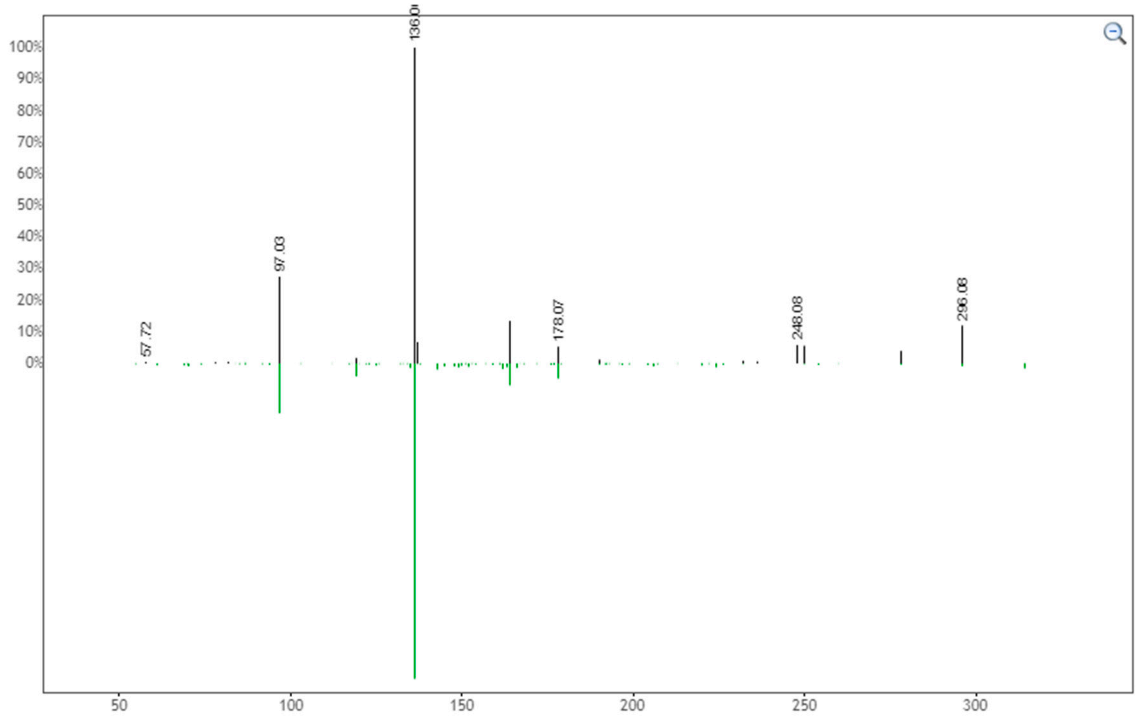

Tyrosine

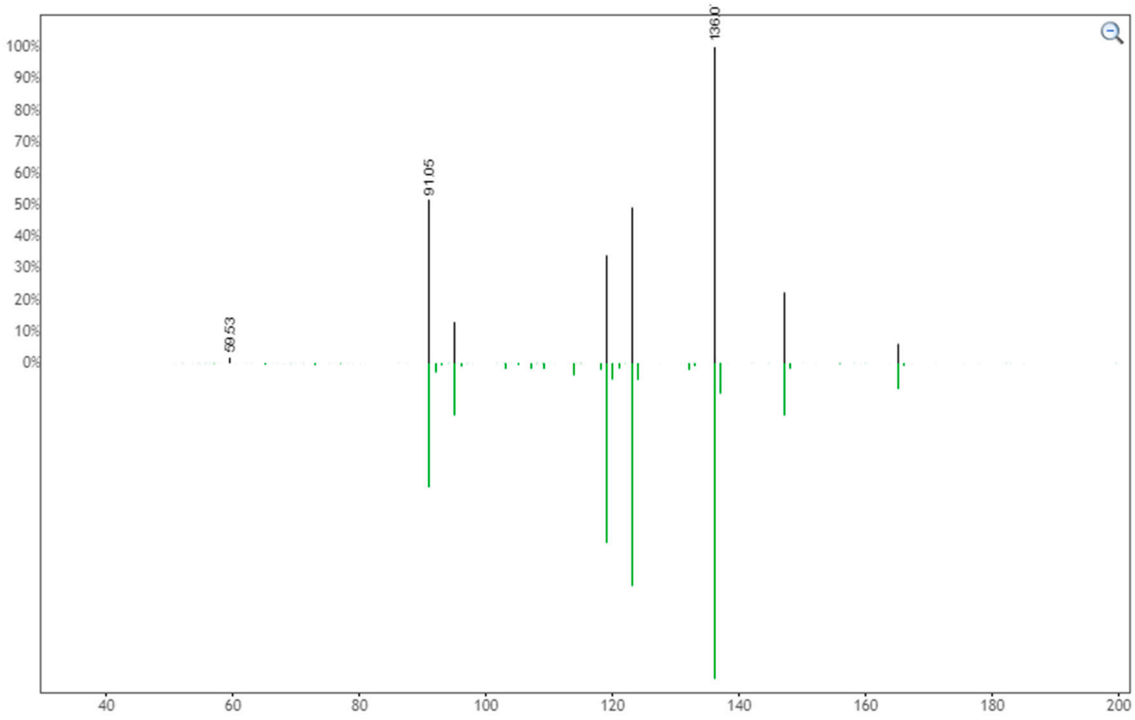

Citric Acid

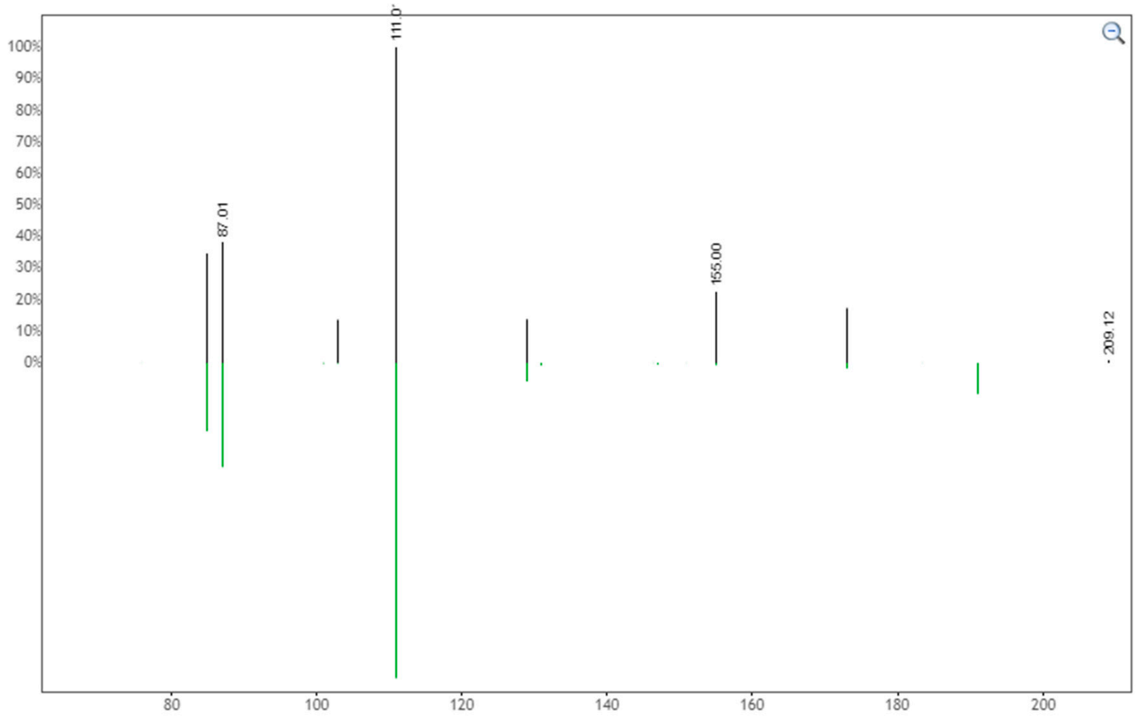

ACon1_000897

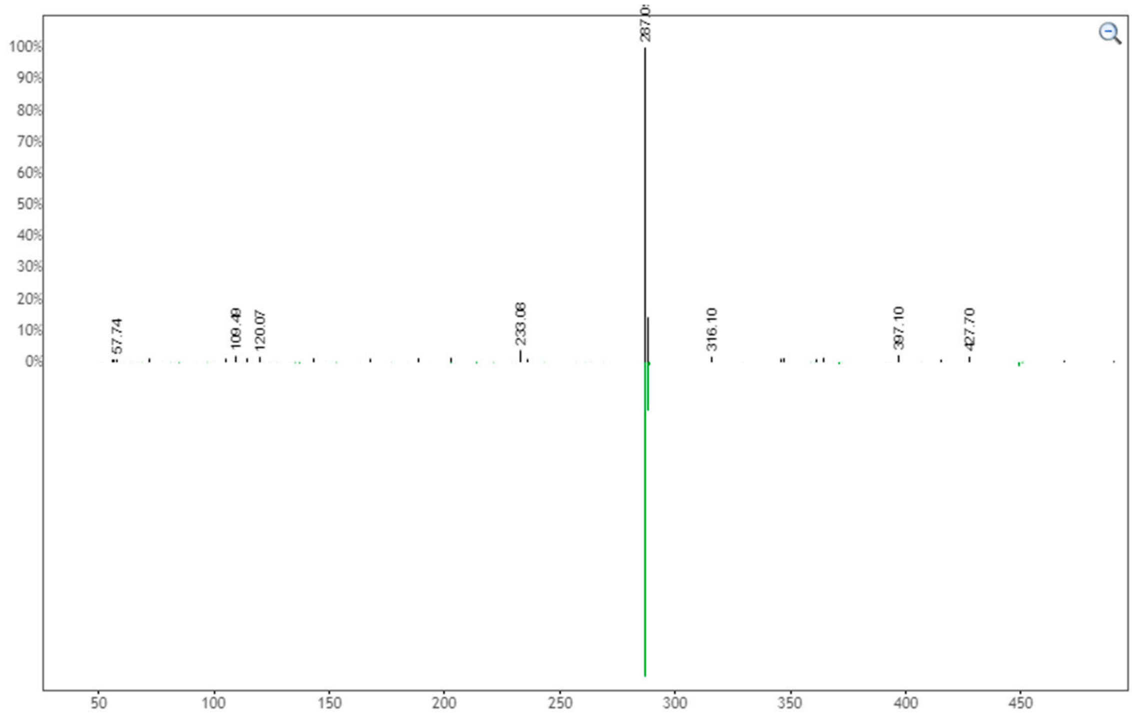

Roseoside

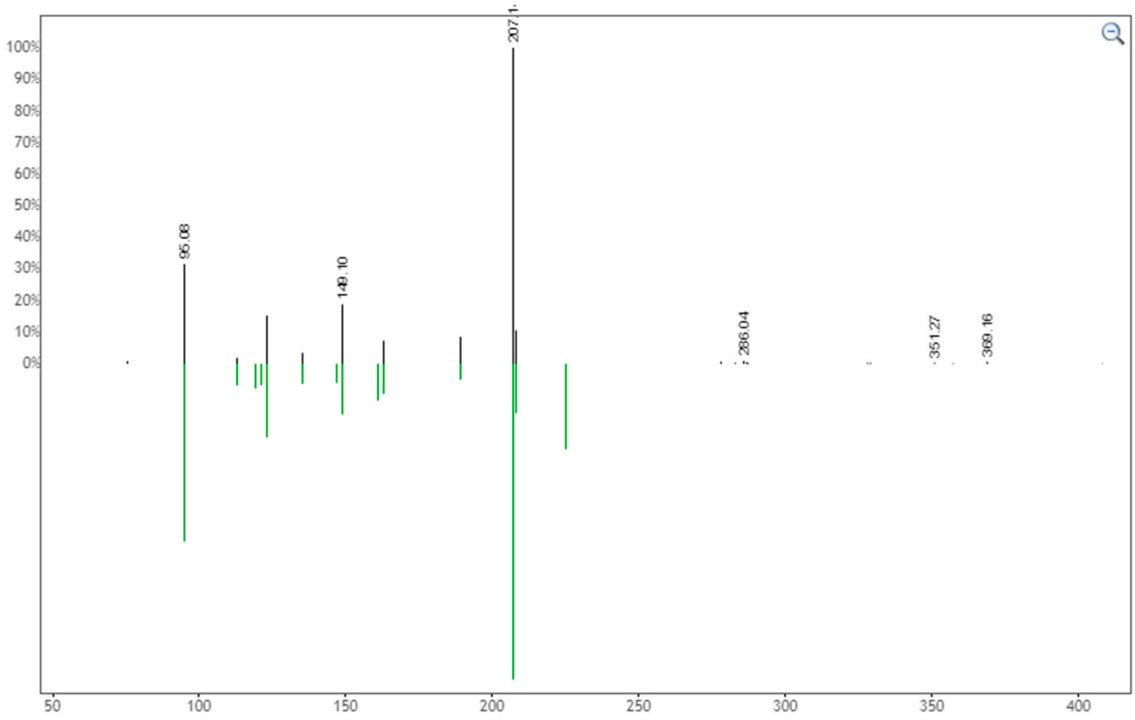

Isoorientin

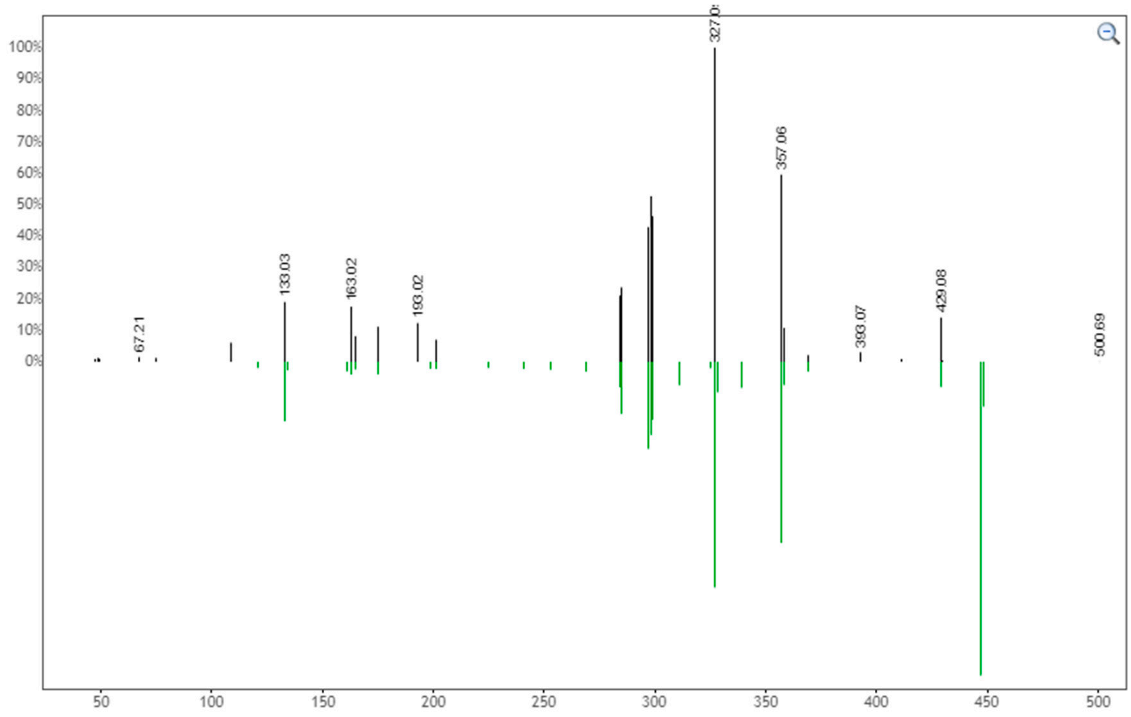

Dihydrokaempferol

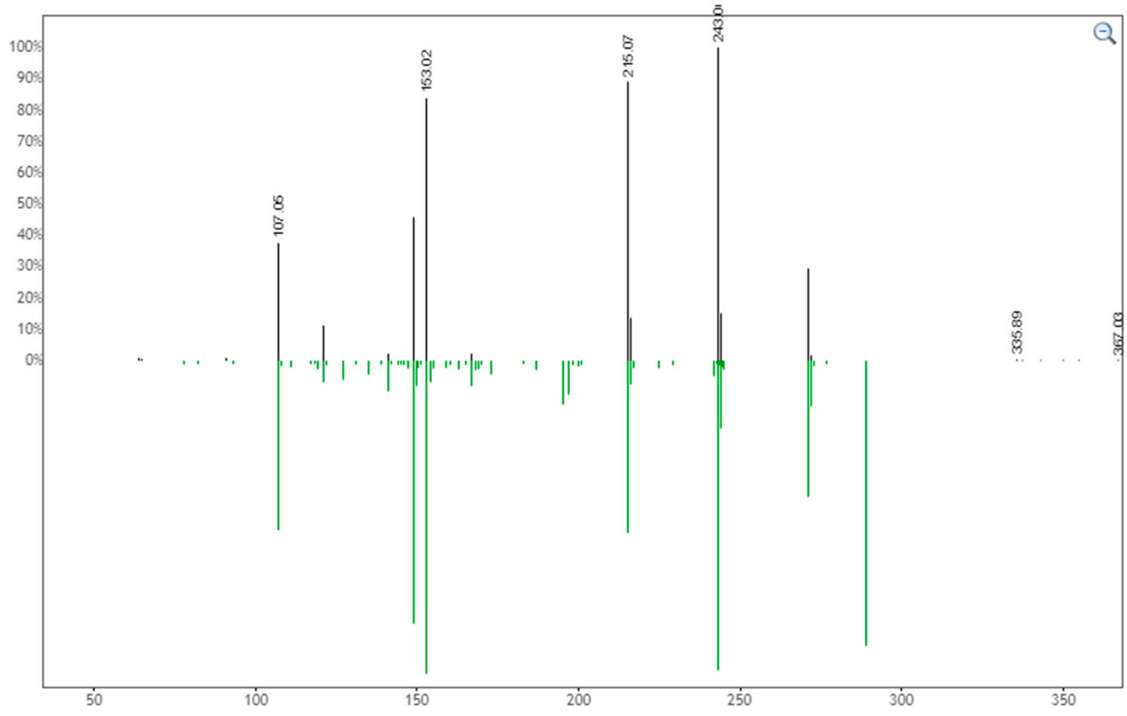

Vitexin-2''-O-rhamnoside

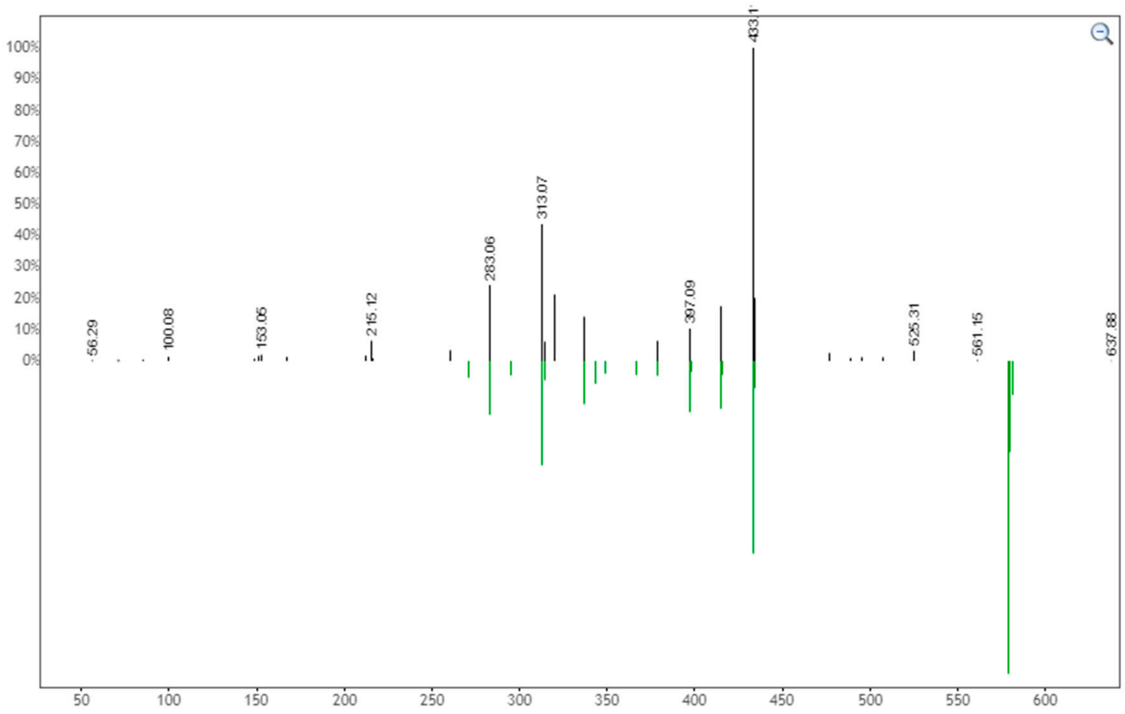

Apigenin-8-C-glucoside

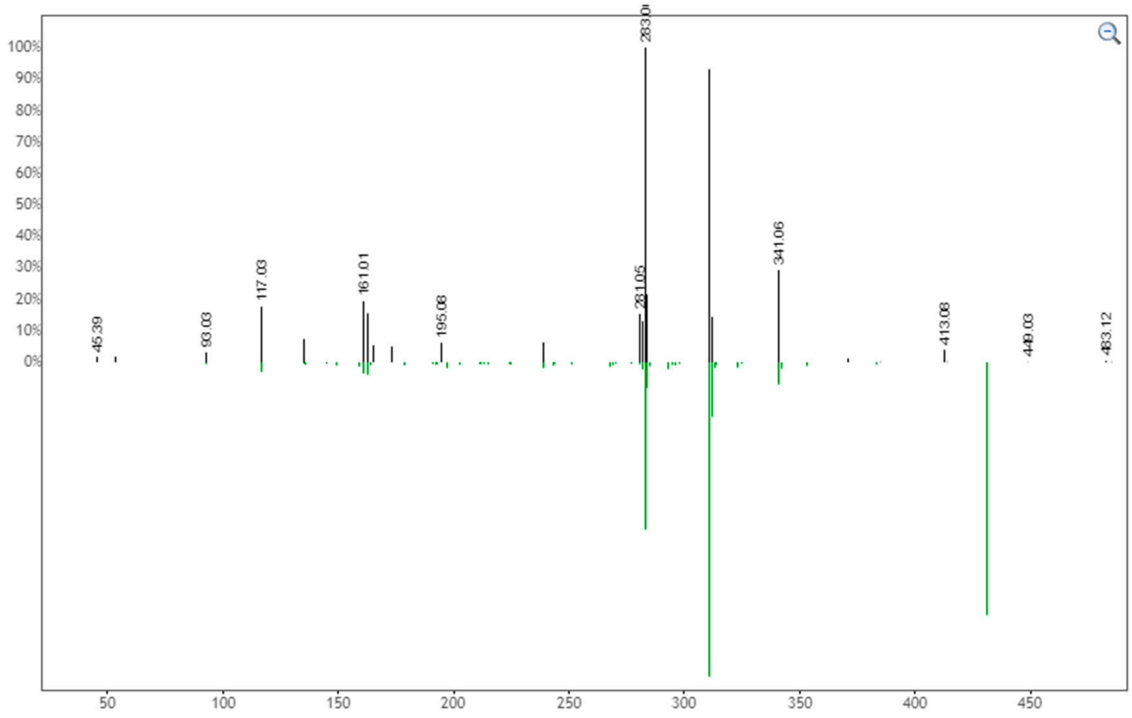

9-(2,3-dihydroxypropoxy)-9-oxononanoic acid

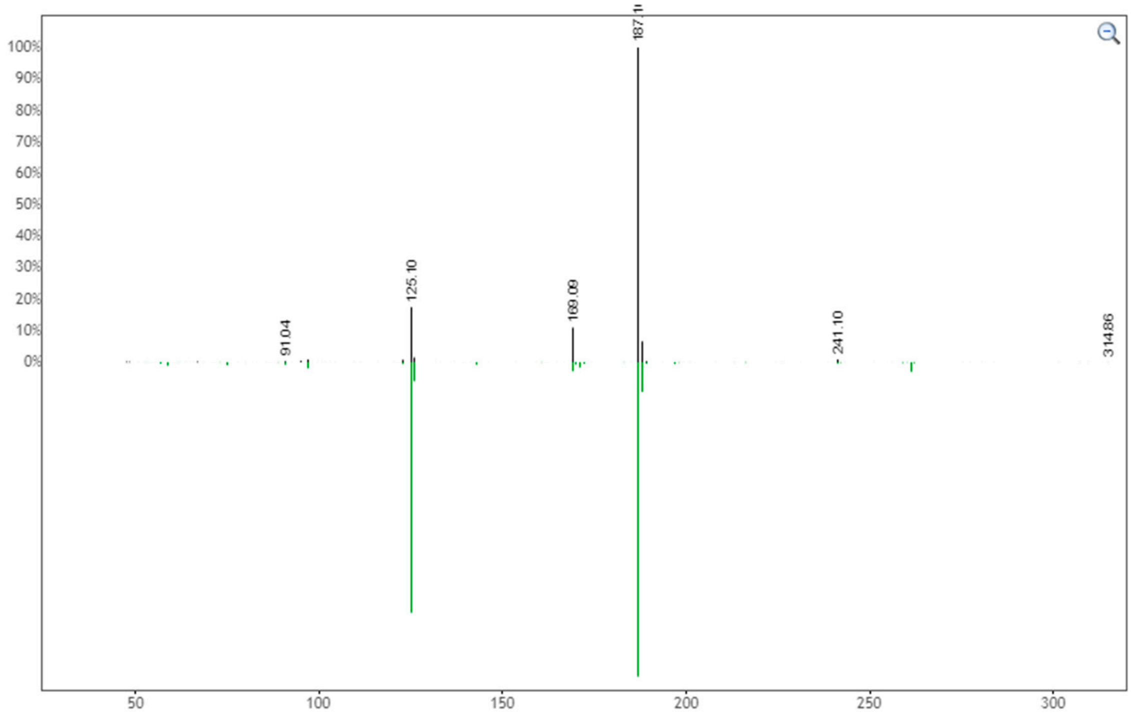

Acanthoside B

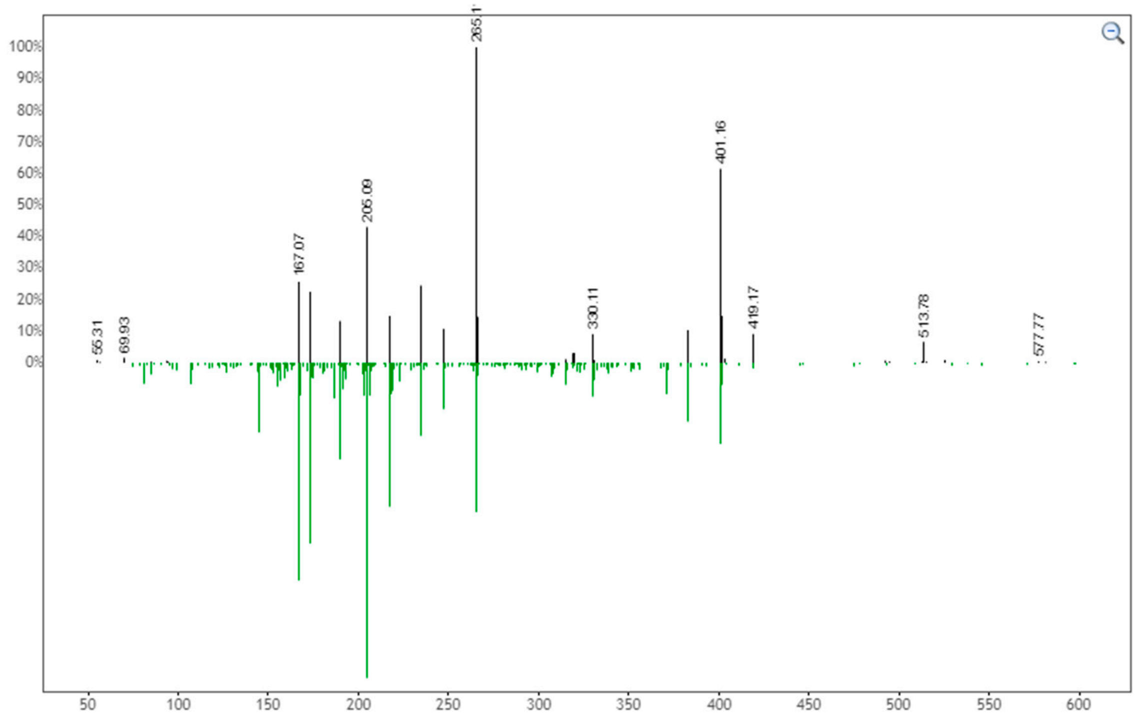

Malvidin 3-O-galactoside

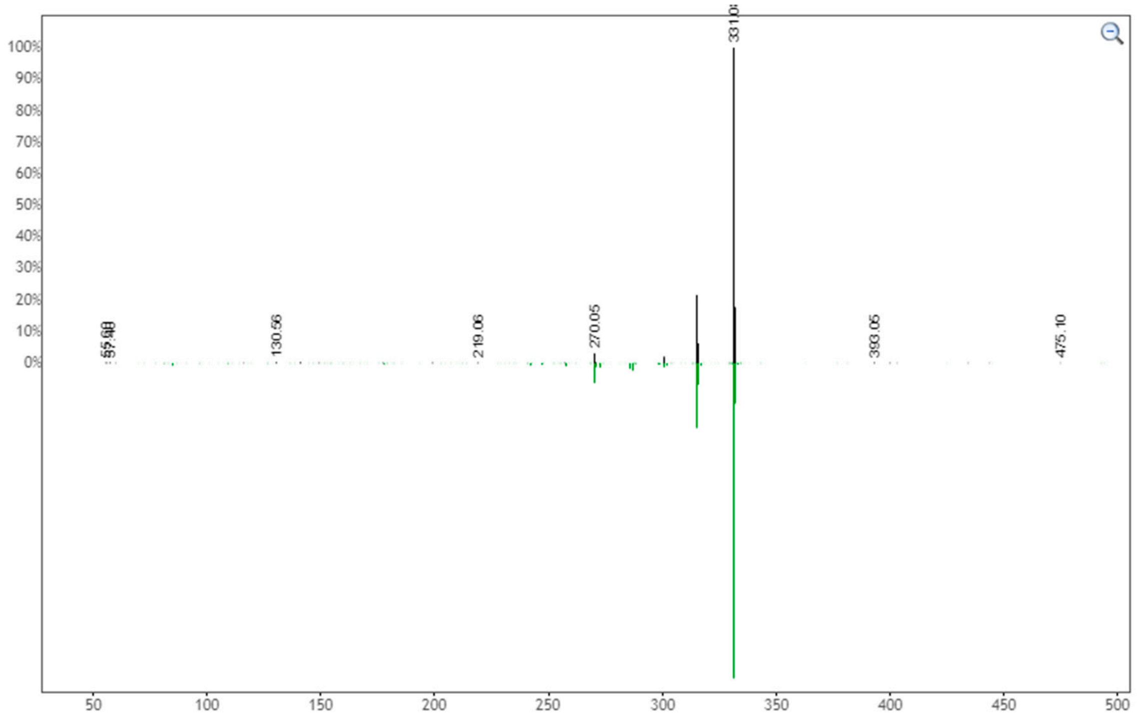

Peonidin 3-O-galactoside

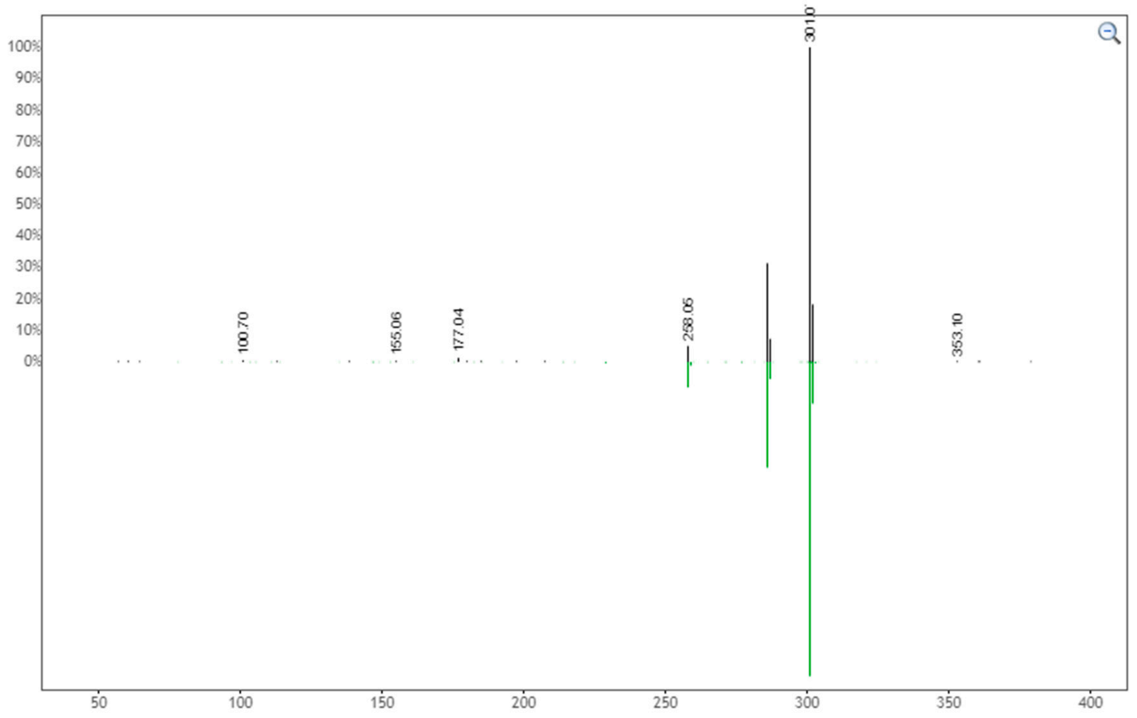

Nonanedioate

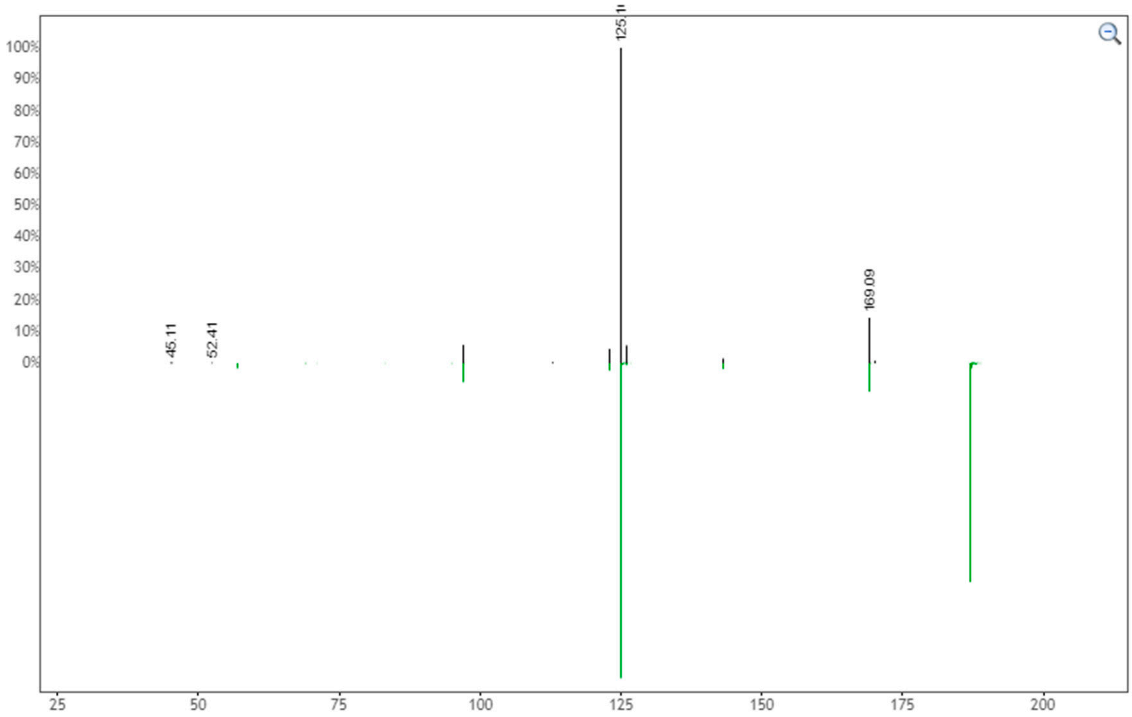

Cinchonine

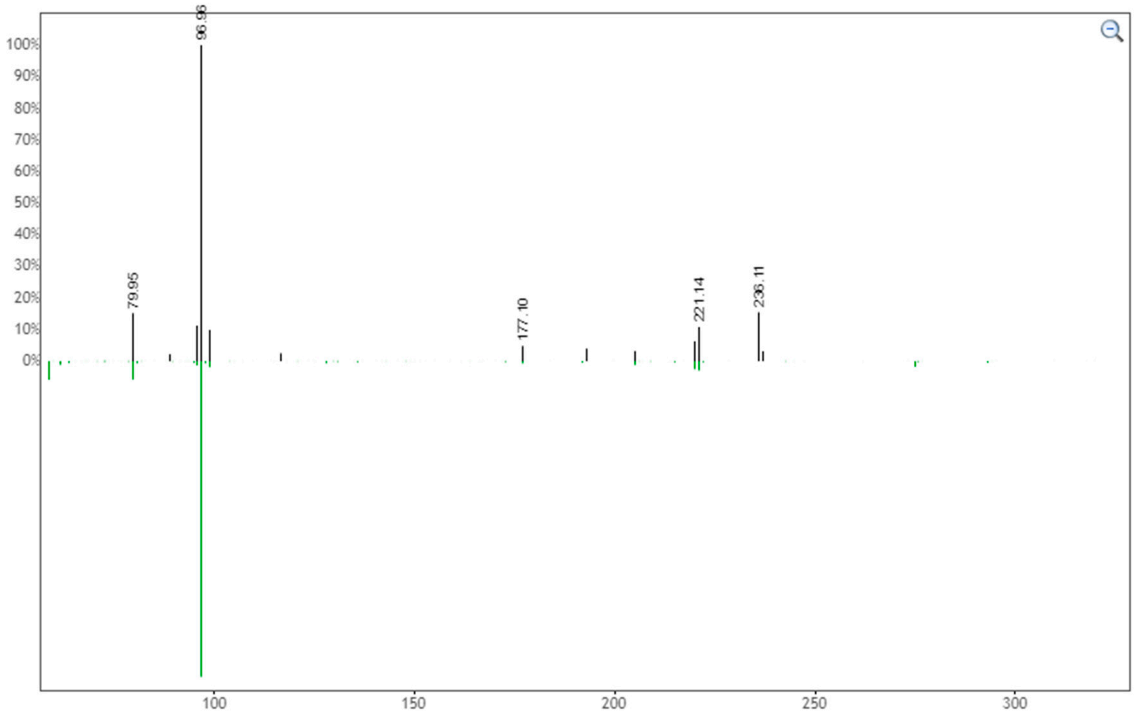

ACon1_002255

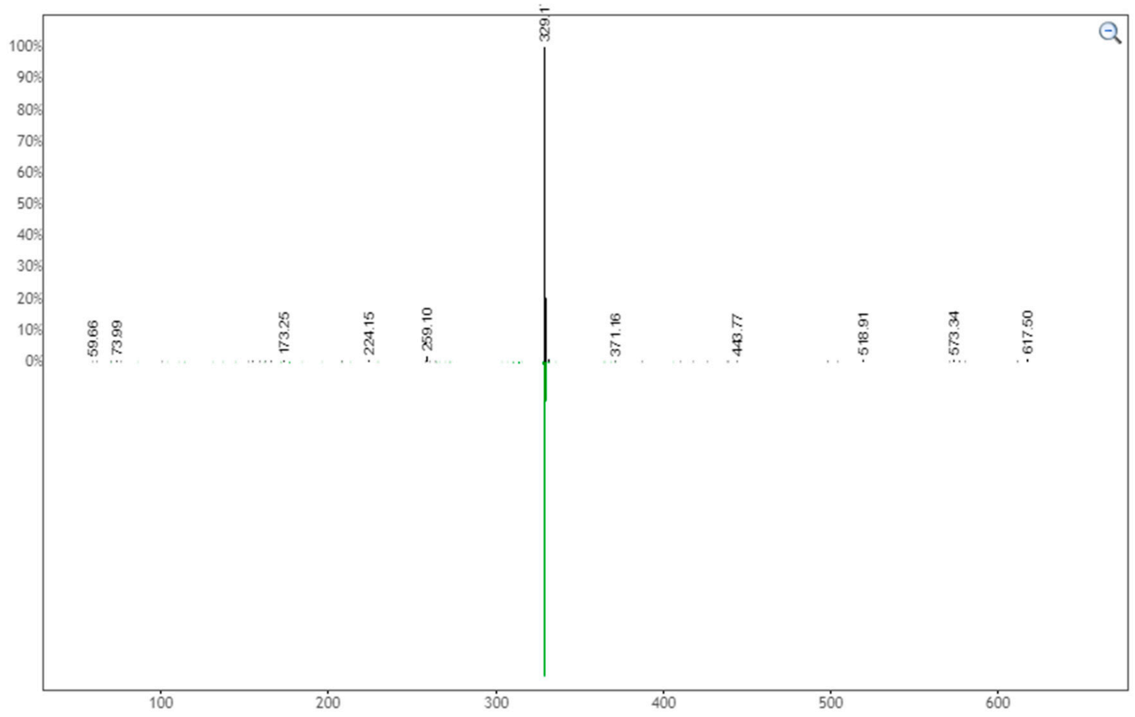

Supplement: Supplementary file 1 [file antioxidants-14-00642-s001.zip › antioxidants-3623124-supplementary.pdf]
